# From Lagrangian Mechanics to Nonequilibrium Thermodynamics: A Variational Perspective

**DOI:** 10.3390/e21010008

**Published:** 2018-12-23

**Authors:** François Gay-Balmaz, Hiroaki Yoshimura

**Affiliations:** 1 Centre National de la Recherche Scientifique (CNRS), Le Laboratoire de Météorologie Dynamique (LMD), Ecole Normale Supérieure, 75005 Paris, France; 2 School of Science and Engineering, Waseda University, Tokyo 169-8050, Japan

**Keywords:** nonequilibrium thermodynamics, variational formulation, nonholonomic constraints, irreversible processes, discrete thermodynamic systems, continuum thermodynamic systems

## Abstract

In this paper, we survey our recent results on the variational formulation of nonequilibrium thermodynamics for the finite-dimensional case of discrete systems, as well as for the infinite-dimensional case of continuum systems. Starting with the fundamental variational principle of classical mechanics, namely, Hamilton’s principle, we show, with the help of thermodynamic systems with gradually increasing complexity, how to systematically extend it to include irreversible processes. In the finite dimensional cases, we treat systems experiencing the irreversible processes of mechanical friction, heat, and mass transfer in both the adiabatically closed cases and open cases. On the continuum side, we illustrate our theory using the example of multicomponent Navier–Stokes–Fourier systems.

## 1. Introduction

This paper reviews our recent work on the development of a variational formulation of nonequilibrium thermodynamics, as established in [1,2,3,4]. This formulation extends to nonequilibrium thermodynamics of the Lagrangian formulation of classical and continuum mechanics that include irreversible processes, such as friction, heat, and mass transfer, chemical reactions, and viscosity.

### 1.1. Some History of the Variational Approaches to Thermodynamics

Thermodynamics was first developed to treat exclusively equilibrium states and the transition from one equilibrium state to another in which a change in temperature plays an important role. In this context, thermodynamics appeared mainly as a theory of heat, and it is viewed today as a branch of *equilibrium thermodynamics*. Such a classical theory, *which does not aim to describe the time evolution* of the system, can be developed in a well-established setting [5] governed by the well-known first and second laws, e.g., [6,7]. It is worth noting that classical mechanics, fluid dynamics, and electromagnetism, being essentially dynamical theories, *cannot* be treated in the context of equilibrium thermodynamics. Although much effort has been applied to the theoretical investigation of nonequilibrium thermodynamics in relation to physics, chemistry, biology, and engineering, the theory of nonequilibrium thermodynamics has not reached the level of completeness. This is in part due to the lack of a general variational formulation for nonequilibrium thermodynamics that would reduce to the classical Lagrangian variational formulation of mechanics in absence of irreversible processes. So far, various variational approaches have been proposed in relation to nonequilibrium thermodynamics. For example, the *principle of least dissipation of energy*, introduced in [8] and later extended in [9,10], underlies the reciprocal relations in linear phenomenological laws, and the *principle of minimum entropy production* by [11,12] sets conditions on steady-state processes. Onsager’s approach was generalized in [13] for systems with nonlinear phenomenological laws. We refer to [14] for reviews and developments of Onsager’s variational principles and for a study of the relation between Onsager’s and Prigogine’s principles. We also refer to Section 6 of [15,16] for overviews on variational approaches to irreversible processes. Note that, however, the variational principles developed in these previous works are not natural extensions of Hamilton’s principle of classical mechanics, because they do not recover Hamilton’s principle for the case in which irreversible processes are not included. Another important work was by [17,18], wherein, in conjunction with thermoelasticity, viscoelasticity, and heat transfer, a *principle of virtual dissipation* as a generalized form of the d’Alembert principle was used with various applications to nonlinear irreversible thermodynamics. In particular, Biot [17] mentioned that the relations between the rate of entropy production and state variables may be given as *nonholonomic constraints*. Nevertheless, this variational approach was restricted to *weakly irreversible systems* or *thermodynamically holonomic and quasi-holonomic* systems. More recently, it was noteworthy that [19] showed a variational formulation for viscoelastic fluids, in which the internal conversion of mechanical power into heat power due to frictional forces was written as a nonholonomic constraint. However, it should be noted that none of the approaches mentioned above present systematic and general variational formulations of nonequilibrium thermodynamics and are hence restricted to a certain class of thermodynamic systems.

Following the initial works of [20,21,22], the geometry of *equilibrium* thermodynamics has been mainly studied via contact geometry by [23], with further developments by [24,25,26]. In this geometric setting, thermodynamic properties are encoded by Legendre submanifolds of the thermodynamic phase space. A step toward a geometric formulation of irreversible processes was made in [27] by lifting port-Hamiltonian systems to the thermodynamic phase space. The underlying geometric structure in this construction is again a contact form. A description of irreversible processes using modifications of Poisson brackets was introduced in [28,29,30]. This was further developed, for instance, in [31,32,33,34,35]. A systematic construction of such brackets from the variational formulation given in the present paper was presented in [36] for the thermodynamics of multicomponent fluids.

### 1.2. Main Features of Our Variational Formulation

The variational formulation for nonequilibrium thermodynamics developed in [1,2,3,4] is distinct from the earlier variational approaches mentioned above, both in its physical meaning and in its mathematical structure, as well as in its goal. Roughly speaking, while most of the earlier variational approaches mainly underlie the equation for the rate of entropy production, in order to justify the expression of the phenomenological laws governing the irreversible processes involved, our variational approach aims to underlie the *complete set of time evolution equations of the system* in such a way that it extends the classical Lagrangian formulation in mechanics to nonequilibrium thermodynamic systems including irreversible processes.

This is accomplished by constructing a generalization of the *Lagrange–d’Alembert principle* of nonholonomic mechanics, where the entropy production of the system, written as the sum of the contribution of each of the irreversible processes, is incorporated into a *nonlinear nonholonomic constraint*. As a consequence, all the *phenomenological laws* are encoded in the nonlinear nonholonomic constraints, to which we naturally associate a *variational constraint* on the allowed variations of the action functional. A natural definition of the variational constraint in terms of the phenomenological constraint is possible thanks to the introduction of the concept of *thermodynamic displacement*, which generalizes the concept of thermal displacement given by [37] to all the irreversible processes.

More concretely, if the system involves internal irreversible processes, denoted by α, and irreversible process at the ports, denoted by β, with thermodynamic fluxes $J_{\alpha },J_{\beta }$ and thermodynamic affinities $X^{\alpha },X^{\beta }$ together with a thermodynamic affinity $X_{\mathrm{ext}}^{\beta }$ associated with the exterior, then the thermodynamic displacements $\Lambda^{\alpha },\Lambda^{\beta }$ are such that ${\dot{\Lambda }}^{\alpha }=X^{\alpha }$ and ${\dot{\Lambda }}^{\beta }=X^{\beta }$. This allows us to formulate the variational constraint associated with the phenomenological constraint in a systematic way, namely, by replacing all the velocities by their corresponding virtual displacement and by removing the external thermodynamic affinity $X_{\mathrm{ext}}^{\beta }$ at the exterior of the system as follows:

$$J_{\alpha }{\dot{\Lambda }}^{\alpha }+J_{\beta }\left({\dot{\Lambda }}^{\beta }-X_{\mathrm{ext}}^{\beta }\right)\;\;⇝\;\;J_{\alpha }\delta \Lambda^{\alpha }+J_{\beta }\delta \Lambda^{\beta }.$$

Our variational formulation thus has a clear and systematic structure that appears to be common for the macroscopic description of the nonequilibrium thermodynamics of physical systems. It can be applied to the finite-dimensional case of discrete systems, such as classical mechanics, electric circuits, chemical reactions, and mass transfer. Further, our variational approach can be naturally extended to the infinite-dimensional case of continuum systems; for instance, it can be applied to some nontrivial example, such as the *multicomponent Navier–Stokes–Fourier equations*. Again, it is emphasized that our variational formulation consistently recovers Hamilton’s principle in classical mechanics when irreversible processes are not taken into account.

### 1.3. Organization of the Paper

In Section 2, we start with a very elementary review of Hamilton’s variational principle in classical mechanics and its extension to the case of mechanical systems with external forces. We also briefly review the variational formulation of mechanical systems with linear nonholonomic constraints by using the Lagrange–d’Alembert principle. Furthermore, we review the extension of Hamilton’s principle to continuum systems and illustrate it with the example of compressible fluids in the Lagrangian description. The variational principle in the Eulerian description is then deduced in the context of *symmetry reduction*. In Section 3, we recall the two laws of thermodynamics as formulated by [38], and we present the variational formulation of nonequilibrium thermodynamics for the finite-dimensional case of discrete systems. We first consider adiabatically closed simple systems and illustrate the variational formulation using the case of a movable piston containing an ideal gas and the case of a system consisting of a chemical species experiencing diffusion between several compartments. We then consider adiabatically closed non-simple systems, such as the adiabatic piston with two cylinders and a system with a chemical species experiencing both diffusion and heat conduction between two compartments. Further, we consider the variational formulation for open systems and illustrate it with the example of a piston device with ports and heat sources. In Section 4, we extend the variational formulation of nonequilibrium thermodynamics to the infinite-dimensional case of continuum systems and consider a multicomponent compressible fluid subject to irreversible processes due to viscosity, heat conduction, and diffusion. The variational formulation is first given in the Lagrangian description, from which the variational formulation in the Eulerian description is deduced. This is illustrated with the multicomponent Navier–Stokes–Fourier equations. In Section 5, we make some concluding remarks and mention further developments based on the variational formulation of nonequilibrium thermodynamics, such as variational discretizations, Dirac structures in thermodynamics, reduction by symmetries, and thermodynamically consistent modeling.

## 2. Variational Principles in Lagrangian Mechanics

### 2.1. Classical Mechanics

One of the most fundamental statements in classical mechanics is the principle of critical action or Hamilton’s principle, according to which the motion of a mechanical system between two given positions is given by a curve that makes the integral of the Lagrangian of the system critical (see, for instance, [39]).

Let us consider a mechanical system with configuration manifold *Q*. For instance, for a system of *N* particles moving in the Euclidean 3-space, the configuration manifold is $Q=R^{3N}$, whereas for a rigid body moving freely in space, $Q=R^{3}\times SO\left(3\right)$, the product of the Euclidean 3-space and the rotation group. Let us denote by $\left(q^{1},\ldots ,q^{n}\right)$ the local coordinates of the manifold *Q*, also known as generalized coordinates of the mechanical system. Let *L* be a given Lagrangian of the system, which usually depends only on the position *q* and velocity *v* of the system and is hence defined on the tangent bundle or *velocity phase space*, TQ, of the manifold *Q*. Recall that *tangent bundle* of a manifold *Q* is the manifold TQ given by the collection of all tangent vectors in *Q*. As a set, it is given by the disjoint union of the *tangent spaces* of *Q*, that is, $TQ={⊔}_{q\in Q}T_{q}Q$, where $T_{q}Q$ is the tangent space to *Q* at *q*. The elements in $T_{q}Q$ are denoted by (q,v). The Lagrangian *L* is usually given by the kinetic minus the potential energy of the system: L(q,v)=K(q,v)−U(q).

Hamilton’s principle is written as follows. Suppose that the system occupies the positions $q_{1}$ and $q_{2}$ at the time $t_{1}$ and $t_{2}$. Then, the motion q(t) of the mechanical system between these two positions is a solution of the critical point condition

(1) $${\left.\frac{d}{d\epsilon }\right|}_{\epsilon =0}\int_{t_{1}}^{t_{2}}L\left(q\left(t,\epsilon \right),\dot{q}\left(t,\epsilon \right)\right)dt=0,$$

where q(t,ϵ), $t\in [t_{1},t_{2}]$, ϵ∈[−a,a], is an arbitrary variation of the curve q(t) with fixed endpoints, i.e., ${q\left(t,\epsilon \right)|}_{\epsilon =0}=q\left(t\right)$ and $q\left(t_{1},\epsilon \right)=q\left(t_{1}\right)$, $q\left(t_{2},\epsilon \right)=q\left(t_{2}\right)$, for all ϵ. The infinitesimal variation associated with a given variation q(t,ϵ) is denoted by

$$\delta q\left(t\right):={\left.\frac{d}{d\epsilon }\right|}_{\epsilon =0}q\left(t,\epsilon \right).$$

From the fixed endpoint conditions, we have $\delta q\left(t_{1}\right)=\delta q\left(t_{2}\right)=0$.

The Hamilton principle in Equation (1) is usually written in short form as

(2) $$\delta \int_{t_{1}}^{t_{2}}L\left(q,\dot{q}\right)dt=0,$$

for arbitrary infinitesimal variations δq, with $\delta q\left(t_{1}\right)=\delta q\left(t_{2}\right)=0$. Throughout this paper, we always use this short notation for the variational principles and also simply refer to δq for variations.

The direct application of Equation (1) gives, in local coordinates $q=(q^{1},\ldots ,q^{n})$,

(3) $$\begin{array}{cc} \delta \int_{t_{1}}^{t_{2}}L\left(q,\dot{q}\right)dt & =\int_{t_{1}}^{t_{2}}\left[\frac{\partial L}{\partial q^{i}}\delta q^{i}+\frac{\partial L}{\partial {\dot{q}}^{i}}\delta {\dot{q}}^{i}\right]dt\\ & =\int_{t_{1}}^{t_{2}}\left[\frac{\partial L}{\partial q^{i}}-\frac{d}{dt}\frac{\partial L}{\partial {\dot{q}}^{i}}\right]\delta q^{i}\;dt+{\left[\frac{\partial L}{\partial {\dot{q}}^{i}}\delta q^{i}\right]}_{t_{1}}^{t_{2}}, \end{array}$$

where Einstein’s summation convention is employed. Since δq is arbitrary and since the boundary term vanishes because of the fixed endpoint conditions, we get from Equation (3) the *Euler–Lagrange equations*:

(4) $$\frac{d}{dt}\frac{\partial L}{\partial {\dot{q}}^{i}}-\frac{\partial L}{\partial q^{i}}=0,\;\;i=1,\ldots ,n.$$

We recall that *L* is called *regular* when the Legendre transform $FL:TQ\to T^{*}Q$, locally given by $\left(q^{i},v^{i}\right)\mapsto \left(q^{i},\frac{\partial L}{\partial v^{i}}\right)$, is a local diffeomorphism, where $T^{*}Q$ denotes the cotangent bundle or *momentum phase space* of *Q*. Recall that cotangent bundle of a manifold *Q* is the manifold $T^{*}Q=\cup_{q\in Q}T_{q}^{*}Q$, where $T_{q}^{*}Q$ is the *cotangent space* at each *q* given as the dual space to $T_{q}Q$. The elements in $T_{q}^{*}Q$ are covectors, denoted by (q,p). When *L* is regular, the Euler–Lagrange Equation (4) yields a second-order differential equation for the curve q(t).

The energy of a mechanical system with the Lagrangian *L* is defined on TQ by

(5) $$E\left(q,v\right)=\langle \frac{\partial L}{\partial v},v\rangle -L\left(q,v\right),$$

where $\langle ,\rangle$ denotes a dual pairing between the elements in $T_{q}^{*}Q$ and $T_{q}Q$. It is easy to check that *E* is conserved along the solutions of the Euler–Lagrange Equation (4), namely,

$$\frac{d}{dt}E\left(q,\dot{q}\right)=\left(\frac{d}{dt}\frac{\partial L}{\partial {\dot{q}}^{i}}-\frac{\partial L}{\partial q^{i}}\right){\dot{q}}^{i}=0.$$

Let us assume that the mechanical system is subject to an external force, given by a map $F^{\mathrm{ext}}:TQ\to T^{*}Q$ assumed to be fiber preserving, i.e., $F^{\mathrm{ext}}\left(q,v\right)\in T_{q}^{*}Q$ for all $\left(q,v\right)\in T_{q}Q$. The extension of Equation (2) to forced mechanical systems is given by

(6) $$\delta \int_{t_{1}}^{t_{2}}L\left(q,\dot{q}\right)dt+\int_{t_{1}}^{t_{2}}\langle F^{\mathrm{ext}}\left(q,\dot{q}\right),\delta q\rangle \;dt=0,$$

for arbitrary variations δq, with $\delta q\left(t_{1}\right)=\delta q\left(t_{2}\right)=0$. The second term in Equation (6) is the time integral of the virtual work $\langle F^{\mathrm{ext}}\left(q,\dot{q}\right),\delta q\rangle$ done by the force field $F^{\mathrm{ext}}:TQ\to T^{*}Q$ with a virtual displacement δq in TQ. The principle in Equation (6) leads to the *forced Euler–Lagrange equations*

(7) $$\frac{d}{dt}\frac{\partial L}{\partial {\dot{q}}^{i}}-\frac{\partial L}{\partial q^{i}}=F_{i}^{\mathrm{ext}}.$$

#### Systems with Nonholonomic Constraints

Hamilton’s principle, as recalled above, is only valid for *holonomic* systems, i.e., systems without constraints or whose constraints are given by functions of the coordinates only, not the velocities. In geometric terms, such constraints are obtained by the specification of a submanifold *N* of the configuration manifold *Q*. In this case, the equations of motion are still given by Hamilton’s principle for the Lagrangian *L* restricted to the tangent bundle TN of the submanifold N⊂Q.

When the constraints cannot be reduced to relations between the coordinates only, they are called *nonholonomic*. Here, we restrict the discussion to nonholonomic constraints that are linear in velocity. Such constraints are locally given in the form

(8) $$\omega_{i}^{\alpha }\left(q\right){\dot{q}}^{i}=0,\;\;\alpha =1,\ldots ,k<n,$$

where $\omega_{i}^{\alpha }$ are functions of local coordinates $q=(q^{1},\ldots ,q^{n})$ on *Q*. Intrinsically, the functions $\omega_{i}^{\alpha }$ are the components of *k* independent one-forms $\omega^{\alpha }$ on *Q*, i.e., $\omega^{\alpha }=\omega_{i}^{\alpha }dq^{i}$, for α=1,…,k. Typical examples of linear nonholonomic constraints are those imposed on the motion of rolling bodies, namely, the velocities of the points in contact should be identical.

For systems with nonholonomic constraints (Equation (8)), the corresponding equations of motion can be derived from a modification of the Hamilton principle called the *Lagrange–d’Alembert principle*, which is given by

(9) $$\delta \int_{t_{1}}^{t_{2}}L\left(q,\dot{q}\right)dt=0,$$

for variations δq subject to the condition

(10) $$\omega_{i}^{\alpha }\left(q\right)\delta q^{i}=0,\;\;\alpha =1,\ldots ,k<n,$$

together with the fixed endpoint conditions $\delta q\left(t_{1}\right)=\delta q\left(t_{2}\right)=0$. Note the occurrence of two constraints with distinct roles. First, there is the constraint in Equation (8) on the solution curve called the *kinematic constraint*. Second, there is the constraint in Equation (10) on the variations used in the principle, referred to as the *variational constraint*. Later, we show that this distinction becomes more noticeable in nonequilibrium thermodynamics.

A direct application of Equations (9) and (10) yields the *Lagrange–d’Alembert equations*

(11) $$\frac{d}{dt}\frac{\partial L}{\partial {\dot{q}}^{i}}-\frac{\partial L}{\partial q^{i}}=\lambda_{\alpha }\omega_{i}^{\alpha }.$$

These equations, together with the constraints in Equation (8), form a complete set of equations for the unknown curves $q^{i}\left(t\right)$ and $\lambda_{\alpha }\left(t\right)$.

For more information on nonholonomic mechanics, the reader can consult [40,41,42]. Note that the Lagrange–d’Alembert principle (9) is *not* a critical curve condition for the action integral *restricted to the space of a curve satisfying the constraints*. Such a principle, which imposes the constraint via a Lagrange multiplier, gives equations that are, in general, not equivalent to the Lagrange–d’Alembert Equation (11), see, e.g., [42,43]. Such equations are sometimes referred to as the vakonomic equations.

### 2.2. Continuum Mechanics

Hamilton’s principle permits a natural extension to continuum systems, such as fluid and elasticity. For such systems, the configuration manifold *Q* is typically a manifold of maps. We shall restrict the discussion here to fluid mechanics in a fixed domain $D\subset R^{3}$ that is assumed to be bounded by a smooth boundary ∂D. Hamilton’s principle for fluid mechanics in the Lagrangian description has been discussed at least since the works of [44] for an incompressible fluid and [45,46] for compressible flows (see also [47] for further references on these early developments). Hamilton’s principle has since then been an important modeling tool in continuum mechanics.

#### 2.2.1. Configuration Manifolds

For fluid mechanics in a fixed domain and before the occurrence of any shocks, the configuration space can be taken as the manifold Q=Diff(D) of diffeomorphisms of D. In this paper, we do not describe the functional analytic setting needed to rigorously work in the framework of infinite dimensional manifolds. For example, one can assume that the diffeomorphisms are of some given Sobolev class, regular enough (at least of class $C^{1}$) so that Diff(D) is a smooth infinite-dimensional manifold and a topological group with a smooth right translation. The tangent bundle to Diff(D) is formally given by the set of vector fields on D covering a diffeomorphism φ and tangent to the boundary, i.e., for each φ∈Diff(D), we have

$$T_{\varphi }\mathrm{Diff}\left(D\right)=\left\{V:D\to TD|V\left(X\right)\in T_{\varphi (X)}D,\;\forall \;X\in D,\;V\left(X\right)\in T_{\varphi (X)}\partial D,\;\forall \;X\in \partial D\right\}.$$

The motion of the fluid is fully described by a curve $\varphi_{t}\in \mathrm{Diff}\left(D\right)$ defining the position $x=\varphi_{t}\left(X\right)$ at time *t* of a fluid particle with label X∈D. The vector field $V_{t}\in T_{\varphi_{t}}\mathrm{Diff}\left(D\right)$ defined by $V_{t}\left(X\right)=\frac{d}{dt}\varphi_{t}\left(X\right)$ is the material velocity of the fluid. In local coordinates, we write $x^{a}=\varphi_{t}^{a}\left(X^{A}\right)$ and $V_{t}^{a}\left(X^{A}\right)=\frac{d}{dt}\varphi_{t}^{a}\left(X^{A}\right)$.

#### 2.2.2. Hamilton’s Principle

Given a Lagrangian L:TQ→R defined on the tangent bundle of the infinite-dimensional manifold Q=Diff(D), Hamilton’s principle formally takes the same form as Equation (2), namely,

(12) $$\delta \int_{t_{1}}^{t_{2}}L\left(\varphi ,\dot{\varphi }\right)dt=0,$$

for variations δφ such that $\delta \varphi_{t_{1}}=\delta \varphi_{t_{2}}=0$.

Let us consider a Lagrangian of the general form

$$L\left(\varphi ,\dot{\varphi }\right)=\int_{D}L\left(\varphi \left(X\right),\dot{\varphi }\left(X\right),\nabla \varphi \left(X\right)\right)d^{3}X,$$

with L being the Lagrangian density and ∇φ being the Jacobian matrix of φ, known as the deformation gradient in continuum mechanics. The variation of the integral yields

$$\begin{array}{cc} \delta \int_{t_{1}}^{t_{2}}L\left(\varphi ,\dot{\varphi }\right)dt & =\int_{t_{1}}^{t_{2}}\;\;\int_{D}\left[\frac{\partial L}{\partial \varphi^{a}}\delta \varphi^{a}+\frac{\partial L}{\partial {\dot{\varphi }}^{a}}\delta {\dot{\varphi }}^{a}+\frac{\partial L}{\partial \varphi_{,A}^{a}}\delta \varphi_{,A}^{a}\right]d^{3}Xdt\\ & =\int_{t_{1}}^{t_{2}}\;\;\int_{D}\left[\frac{\partial L}{\partial \varphi^{a}}\delta \varphi^{a}-\frac{\partial }{\partial t}\frac{\partial L}{\partial {\dot{\varphi }}^{a}}-\frac{\partial }{\partial A}\frac{\partial L}{\partial \varphi_{,A}^{a}}\right]\delta \varphi^{a}d^{3}Xdt\\ & \;\;+\int_{D}{\left[\frac{\partial L}{\partial {\dot{\varphi }}^{a}}\delta \varphi^{a}\right]}_{t_{1}}^{t_{2}}d^{3}X+\int_{t_{1}}^{t_{2}}\;\;\int_{\partial D}\frac{\partial L}{\partial \varphi_{,A}^{a}}N_{A}\delta \varphi^{a}dSdt, \end{array}$$

where N is the outward-pointing unit normal vector field to the boundary ∂D, and dS denotes the area element on the surface ∂D. Hamilton’s principle thus yields the Euler–Lagrange equations and the boundary condition

(13) $$\frac{\partial }{\partial t}\frac{\partial L}{\partial \dot{\varphi }}+\mathrm{DIV}\frac{\partial L}{\partial \nabla \varphi }=\frac{\partial L}{\partial \varphi }\;\mathrm{and}\;{\left.\frac{\partial L}{\partial \nabla \varphi }\cdot N\right|}_{T\partial D}=0\;\;\mathrm{on}\;\partial D,$$

where the divergence operator is defined as ${\left(\mathrm{DIV}\frac{\partial L}{\partial \nabla \varphi }\right)}_{a}=\frac{\partial }{\partial A}\frac{\partial L}{\partial \varphi_{,A}^{a}}$. The tensor field

(14) $$P:=-\frac{\partial L}{\partial \nabla \varphi },\;i.e.\;P_{a}^{A}=-\frac{\partial L}{\partial \varphi_{,A}^{a}}$$

is called the *first Piola–Kirchhoff stress tensor* (see, e.g., [48]).

#### 2.2.3. The Lagrangian of the Compressible Fluid

For a compressible fluid, the Lagrangian has the standard form

(15) $$\begin{array}{cc} L(\varphi ,\dot{\varphi }) & =K\left(\varphi ,\dot{\varphi }\right)-U\left(\varphi \right)=\int_{D}\left[\frac{1}{2}\varrho_{\mathrm{ref}}\left(X\right){\left|\dot{\varphi }\left(X\right)\right|}^{2}-E\left(\varrho_{\mathrm{ref}}\left(X\right),S_{\mathrm{ref}}\left(X\right),\nabla \varphi \left(X\right)\right)\right]d^{3}X, \end{array}$$

with $\varrho_{\mathrm{ref}}\left(X\right)$ and $S_{\mathrm{ref}}\left(X\right)$ being the mass density and entropy density in the reference configuration. The two terms in Equation (15) are, respectively, the total kinetic energy of the fluid and minus the total internal energy of the fluid. The function E is a general expression for the internal energy density written in terms of $\varrho_{\mathrm{ref}}\left(X\right)$, $S_{\mathrm{ref}}\left(X\right)$, and the deformation gradient ∇φ(X). For fluids, E depends on the deformation gradient only through the Jacobian of φ, denoted by $J_{\varphi }$. This fact is compatible with the material covariance property of E, written as

(16) $$E\left(\psi^{*}\varrho_{\mathrm{ref}},\psi^{*}S_{\mathrm{ref}},\nabla \left(\varphi \circ \psi \right)\right)=\psi^{*}\left[E\left(\varrho_{\mathrm{ref}},S_{\mathrm{ref}},\nabla \varphi \right)\right],\;\mathrm{for}\;\mathrm{all}\;\psi \in \mathrm{Diff}(D),$$

where the pull-back notation is defined as

(17) $$\varphi^{*}f=\left(f\circ \varphi \right)J_{\varphi }$$

for some function *f* defined on D. From Equation (16), we deduce the existence of a function ϵ such that

(18) $$E\left(\varrho_{\mathrm{ref}},S_{\mathrm{ref}},\nabla \varphi \right)=\varphi^{*}\left[\epsilon (\rho ,s)\right],\;\mathrm{for}\;\rho =\varphi_{*}\varrho_{\mathrm{ref}},\;s=\varphi_{*}S_{\mathrm{ref}},$$

(see [48,49]). The function ϵ=ϵ(ρ,s) is the internal energy density in the spatial description expressed in terms of the mass density ρ and entropy density *s*.

For the Lagrangian Equation (15) and with the assumption in Equation (16), the first Piola–Kirchhoff stress tensor (Equation (14)) and its divergence are computed as

(19) $$P_{a}^{A}=\frac{\partial E}{\partial \varphi_{,A}^{a}}=-pJ_{\varphi }{\left(\varphi^{-1}\right)}_{,a}^{A}\;\mathrm{and}\;\mathrm{DIV}P=\left(\nabla p\circ \varphi \right)J_{\varphi },$$

where $p=\frac{\partial \epsilon }{\partial \rho }\rho +\frac{\partial \epsilon }{\partial s}s-\epsilon$ is the pressure. Note that for all $\delta \varphi^{a}$ parallel to the boundary, we have $P_{a}^{A}N_{A}\delta \varphi^{a}=-pJ_{\varphi }{\left(\varphi^{-1}\right)}_{,a}^{A}N_{A}\delta \varphi^{a}=0$, since ${\left(\varphi^{-1}\right)}_{,a}^{A}\delta \varphi^{a}$ is parallel to the boundary. Hence, the boundary condition in Equation (13) is always satisfied. From Equation (19), the Euler–Lagrange Equation (13) becomes

(20) $$\varrho_{\mathrm{ref}}\ddot{\varphi }=\left(\nabla p\circ \varphi \right)J_{\varphi }.$$

Equation (20) is the equation of motion for a compressible fluid in the *material (or Lagrangian) description*, which directly follows from the Hamilton principle in Equation (12) applied to the Lagrangian Equation (15). It is, however, highly desirable to have a variational formulation that directly produces the equations of motion in the standard spatial (or Eulerian) description. This is recalled below in Section 2.3 by using Lagrangian reduction by symmetry.

### 2.3. Lagrangian Reduction by Symmetry

When symmetry is available in a mechanical system, it is often possible to exploit it in order to reduce the dimension of the system and thereby facilitate its study. This process, called *reduction by symmetry*, is presently well understood on both the Lagrangian and Hamiltonian sides (see [50] for an introduction and references).

On the Hamiltonian side, this process is based on the reduction of symplectic or Poisson structures while, on the Lagrangian side, it is usually based on the reduction of variational principles (see [51,52,53]). Consider a mechanical system with a configuration manifold *Q* and Lagrangian L:TQ→R, and consider also the action of a Lie group *G* on *Q*, denoted here simply as q↦g·q for g∈G, q∈Q. This action naturally induces an action on the tangent bundle TQ, denoted here simply as (q,v)↦(g·q,g·v), called the *tangent-lifted action*. We say that the action is a symmetry for the mechanical system if the Lagrangian *L* is invariant under this tangent-lifted action. In this case, *L* induces a *symmetry-reduced Lagrangian*ℓ:(TQ)/G→R defined on the quotient space (TQ)/G of the tangent bundle with respect to the action. The goal of the Lagrangian reduction process is to derive the equations of motion directly on the reduced space (TQ)/G. Under standard hypotheses on the action, this quotient space is a manifold, and one obtains the *reduced Euler–Lagrange equations* by computing the *reduced variational principle* for the action integral $\int_{t_{1}}^{t_{2}}\ell \;dt$ induced by Hamilton’s principle (Equation (2)) for the action integral $\int_{t_{1}}^{t_{2}}L\;\mathrm{dt}$. The main difference between the reduced variational principle and Hamilton’s principle is the occurrence of constraints on the variations to be considered when computing the critical curves for $\int_{t_{1}}^{t_{2}}\ell \;dt$. These constraints are uniquely associated with the reduced character of the variational principle and are not due to physical constraints as in Equation (10) earlier.

We now quickly recall the application of Lagrangian reduction for the treatment of fluid mechanics in a fixed domain (see Section 2.2) by following the Euler–Poincaré reduction approach in [54]. In this case, the Lagrangian reduction process encodes the shift from the material (or Lagrangian) description to the spatial (or Eulerian) description.

As we recalled above, in the material description, the motion of the fluid is described by a curve of diffeomorphisms $\varphi_{t}$ in the configuration manifold Q=Diff(D), and the evolution Equation (20) for $\varphi_{t}$ follows from the standard Hamilton principle.

In the spatial description, the dynamics are described by the Eulerian velocity v(t,x), the mass density ρ(t,x) and the entropy density s(t,x), defined in terms of $\varphi_{t}$ as

(21) $$v_{t}={\dot{\varphi }}_{t}\circ \varphi_{t}^{-1},\;\rho_{t}={\left(\varphi_{t}\right)}_{*}\varrho_{\mathrm{ref}},\;s_{t}={\left(\varphi_{t}\right)}_{*}S_{\mathrm{ref}}.$$

Using these relations and Equation (18), the Lagrangian Equation (15) in the material description induces the following expression in the spatial description:

$$\ell \left(v,\rho ,s\right)=\int_{D}\left[\frac{1}{2}\rho {\left|v\right|}^{2}-\varepsilon \left(\rho ,s\right)\right]d^{3}x.$$

The symmetry group underlying the Lagrangian reduction process is the subgroup

$$\mathrm{Diff}{\left(D\right)}_{\varrho_{\mathrm{red}},S_{\mathrm{ref}}}\subset \mathrm{Diff}\left(D\right)$$

of diffeomorphisms that preserve both the mass density $\varrho_{\mathrm{ref}}$ and entropy density $S_{\mathrm{ref}}$ in the reference configuration. So, we have Q=Diff(D) and $G=\mathrm{Diff}{\left(D\right)}_{\varrho_{\mathrm{red}},S_{\mathrm{ref}}}$ in the general Lagrangian reduction setting described above.

From the relations in Equation (21), we deduce that the variations δφ used in Hamilton’s principle in Equation (12) induce the variations

(22) $$\delta v=\partial_{t}\zeta +v\cdot \nabla \zeta -\zeta \cdot \nabla v,\;\delta \rho =-\mathrm{div}\left(\rho \zeta \right),\;\delta s=-\mathrm{div}\left(s\zeta \right),$$

where $\zeta =\delta \varphi \circ \varphi^{-1}$ is an arbitrary time-dependent vector field parallel to ∂D. From Lagrangian reduction theory, the Hamilton principle in Equation (12) induces, in the Eulerian description, the (reduced) variational principle

(23) $$\delta \int_{t_{1}}^{t_{2}}\ell \left(v,\rho ,s\right)\;dt=0,$$

for variations δv, δρ, δs constrained by the relations in Equation (22) with $\zeta \left(t_{1}\right)=\zeta \left(t_{2}\right)=0$. This principle yields the compressible fluid equations $\rho (\partial_{t}v+v\cdot \nabla v)=-\nabla p$ in the Eulerian description, while the continuity equations $\partial_{t}\rho +\mathrm{div}\left(\rho v\right)=0$ and $\partial_{t}s+\mathrm{div}\left(sv\right)=0$ follow from the definition of ρ and *s* in Equation (21) (see [54]). We refer to [49] for an extension of this Lagrangian reduction approach to the case of fluids with a free boundary.

The variational formulations in Equations (22) and (23) are extended in Section 4 to include irreversible processes and are illustrated using the Navier–Stokes–Fourier system as an example.

## 3. Variational Formulation for Discrete Thermodynamic Systems

In this section, we present a variational formulation for the finite-dimensional case of discrete thermodynamic systems that reduces to Hamilton’s variational principle in Equation (2) in absence of irreversible processes. The form of this variational formulation is similar to that of nonholonomic mechanics recalled earlier (see Equations (8)–(10)) in the sense that the critical curve condition is subject to two constraints: a *kinematic constraint* on the solution curve and a *variational constraint* on the variations to be considered when computing the criticality condition. A major difference, however, with the Lagrange–d’Alembert principle recalled above is that the constraints are *nonlinear* in velocity. This formulation is extended to continuum systems in Section 4.

Before presenting the variational formulation, we recall below the two laws of thermodynamics as formulated in [38].

- *The two laws of thermodynamics*

Let us denote by Σ a physical system and by $\Sigma^{\mathrm{ext}}$ its exterior. The state of the system is defined by a set of mechanical variables and a set of thermal variables. State functions are functions of these variables. Stueckelberg’s formulation of the two laws is given as follows.

- *First law:*

For every system Σ, there exists an extensive scalar state function *E*, called *energy*, which satisfies

$$\frac{d}{dt}E\left(t\right)=P_{W}^{\mathrm{ext}}\left(t\right)+P_{H}^{\mathrm{ext}}\left(t\right)+P_{M}^{\mathrm{ext}}\left(t\right),$$

where $P_{W}^{\mathrm{ext}}$ is the *power associated with the work done on the system* (here, *work* includes not only *mechanical work* by the action of forces but also other physical work, such as that by the action of electric voltages, etc.), $P_{H}^{\mathrm{ext}}$ is the *power associated with the transfer of heat into the system*, and $P_{M}^{\mathrm{ext}}$ is the *power associated with the transfer of matter into the system.* As we recall below, a transfer of matter into the system is associated with a transfer of work and heat. By convention, $P_{W}^{\mathrm{ext}}$ and $P_{H}^{\mathrm{ext}}$ denote uniquely the power associated with a transfer of work and heat into the system that is *not associated with a transfer of matter*. The power associated with a transfer of heat or work due to a transfer of matter is included in $P_{M}^{\mathrm{ext}}$.

Given a *thermodynamic system*, the following terminology is generally adopted:

- A system is said to be *closed* if there is no exchange of matter, i.e., $P_{M}^{\mathrm{ext}}\left(t\right)=0$. When $P_{M}^{\mathrm{ext}}\left(t\right)\neq 0$, the system is said to be *open*.
- A system is said to be *adiabatically closed* if it is closed and there are no heat exchanges, i.e., $P_{M}^{\mathrm{ext}}\left(t\right)=P_{H}^{\mathrm{ext}}\left(t\right)=0$.
- A system is said to be *isolated* if it is adiabatically closed and there is no mechanical power exchange, i.e., $P_{M}^{\mathrm{ext}}\left(t\right)=P_{H}^{\mathrm{ext}}\left(t\right)=P_{W}^{\mathrm{ext}}\left(t\right)=0$.

From the first law, it follows that the *energy of an isolated system is constant*.

- *Second law:*

For every system Σ, there exists an extensive scalar state function *S*, called *entropy*, which obeys the following two conditions

(a) Evolution part:
  If the system is adiabatically closed, the entropy *S* is a non-decreasing function with respect to time, i.e.,
  $$\frac{d}{dt}S\left(t\right)=I\left(t\right)\geq 0,$$
  where I(t) is the *entropy production rate* of the system accounting for the irreversibility of internal processes.
(b) Equilibrium part:
  If the system is isolated, as time tends to infinity, the entropy tends toward a finite local maximum of the function *S* over all thermodynamic states ρ compatible with the system, i.e.,
  $$\lim_{t\to +\infty }S\left(t\right)=\max_{\rho \;\mathrm{compatible}}S\left[\rho \right].$$

By definition, the evolution of an isolated system is said to be *reversible* if I(t)=0, namely, the entropy is constant. In general, the evolution of a system Σ is said to be *reversible* if the evolution of the total isolated system with which Σ interacts is reversible.

Based on this formulation of the two laws, Stueckelberg and Scheurer [38] developed a systematic approach for the derivation of the equations of motion for thermodynamic systems; it is especially well suited for the understanding of nonequilibrium thermodynamics as an extension of classical mechanics. We refer, for instance, to [55,56,57] for the applications of Stueckelberg’s approach to the derivation of equations of motion for thermodynamical systems.

We present our approach by considering systems with gradually increasing level of complexity. First we treat *adiabatically closed* systems that have only one entropy variable or, equivalently, one temperature. Such systems, called *simple systems*, may involve the irreversible processes of mechanical friction and internal matter transfer. Then, we treat a more general class of finite-dimensional adiabatically closed thermodynamic systems with several entropy variables, which may also involve the irreversible process of heat conduction. We then consider *open* finite-dimensional thermodynamic systems, which can exchange heat and matter with the exterior. Finally, we explain how chemical reactions can be included in the variational formulation.

### 3.1. Adiabatically Closed Simple Thermodynamic Systems

We present below the definition of finite-dimensional and simple systems following [38]. A *finite-dimensional thermodynamic system* Σ is a collection $\Sigma =\cup_{A=1}^{P}\Sigma_{A}$ of a finite number of interacting simple thermodynamic systems $\Sigma_{A}$. By definition, a *simple thermodynamic system* is a macroscopic system for which one (scalar) thermal variable and a finite set of nonthermal variables are sufficient to entirely describe the state of the system. From the second law of thermodynamics, we can always choose the entropy *S* as a thermal variable. A typical example of such a simple system is the one-cylinder problem. We refer to [55] for a systematic treatment of this system via Stueckelberg’s approach.

#### 3.1.1. Variational Formulation for Mechanical Systems with Friction

We consider here a simple system which can be described only by a single entropy as a thermodynamic variable, besides mechanical variables. As in Section 2.1 above, let *Q* be the configuration manifold associated with the mechanical variables of the simple system. The Lagrangian of the simple thermodynamic system is thus a function:

L:TQ×R→R,(q,v,S)↦L(q,v,S),

where S∈R is the entropy. We assume that the system involves external and friction forces given by fiber-preserving maps $F^{\mathrm{ext}},F^{\mathrm{fr}}:TQ\times R\to T^{*}Q$, i.e., such that $F^{\mathrm{fr}}\left(q,v,S\right)\in T_{q}^{*}Q$, similar to $F^{\mathrm{ext}}$. As stated in [1], the variational formulation for this simple system is given as follows:

Find the curves q(t), S(t) which are critical for the *variational condition*

(24) $$\delta \int_{t_{1}}^{t_{2}}L\left(q,\dot{q},S\right)dt+\int_{t_{1}}^{t_{2}}\langle F^{\mathrm{ext}}\left(q,\dot{q},S\right),\delta q\rangle \;dt=0,$$

subject to the *phenomenological constraint*

(25) $$\frac{\partial L}{\partial S}\left(q,\dot{q},S\right)\dot{S}=\langle F^{\mathrm{fr}}\left(q,\dot{q},S\right),\dot{q}\rangle ,\;$$

and for variations subject to the *variational constraint*

(26) $$\frac{\partial L}{\partial S}\left(q,\dot{q},S\right)\delta S=\langle F^{\mathrm{fr}}\left(q,\dot{q},S\right),\delta q\rangle ,$$

with $\delta q\left(t_{1}\right)=\delta q\left(t_{2}\right)=0$.

Taking variations of the integral in Equation (24), integrating by parts, and using $\delta q\left(t_{1}\right)=\delta \left(t_{2}\right)=0$, it follows that

$$\int_{t_{1}}^{t_{2}}\left[\left(\frac{\partial L}{\partial q^{i}}-\frac{d}{dt}\frac{\partial L}{\partial {\dot{q}}^{i}}+F_{i}^{\mathrm{ext}}\right)\delta q^{i}+\frac{\partial L}{\partial S}\delta S\right]\;dt.$$

From the variational constraint in Equation (26), the last term in the integrand of the above equation can be replaced by $F_{i}^{\mathrm{fr}}\delta q^{i}$. Hence, using Equation (25), we get the following system of evolution equations for the curves q(t) and S(t):

(27) $$\left\{\begin{array}{c} \;\frac{d}{dt}\frac{\partial L}{\partial \dot{q}}-\frac{\partial L}{\partial q}=F^{\mathrm{fr}}\left(q,\dot{q},S\right)+F^{\mathrm{ext}}\left(q,\dot{q},S\right),\\ \frac{\partial L}{\partial S}\dot{S}=\langle F^{\mathrm{fr}}\left(q,\dot{q},S\right),\dot{q}\rangle . \end{array}\right.$$

This variational formulation is a generalization of Hamilton’s principle in Lagrangian mechanics in the sense that it can yield irreversible processes in addition to the Lagrange–d’Alembert equations with external and friction forces. In this generalized variational formulation, the temperature is defined as minus the derivative of *L* with respect to *S*, i.e., $T=-\frac{\partial L}{\partial S}$, which is assumed to be positive. When the Lagrangian has the standard form

L(q,v,S)=K(q,v)−U(q,S),

where the kinetic energy *K* is assumed to be independent of *S*, and U(q,S) is the internal energy, then $T=-\frac{\partial L}{\partial S}=\frac{\partial U}{\partial S}$ recovers the standard definition of the temperature in thermodynamics.

When the friction force vanishes, the entropy is constant from the second equation in Equation (27), and hence, the system in Equation (27) reduces to the forced Euler–Lagrange equations in classical mechanics for a Lagrangian depending parametrically on a given constant entropy $S_{0}$.

The total energy associated with the Lagrangian is still defined by the same expression as in Equation (5) except that it now depends on *S*, i.e., we define the total energy E:TQ×R→R by

(28) $$E\left(q,v,S\right)=\langle \frac{\partial L}{\partial v},v\rangle -L\left(q,v,S\right).$$

Along the solution curve of Equation (27), we have

$$\frac{d}{dt}E=\left(\frac{d}{dt}\frac{\partial L}{\partial {\dot{q}}^{i}}-\frac{\partial L}{\partial q^{i}}\right){\dot{q}}^{i}-\frac{\partial L}{\partial S}\dot{S}=F_{i}^{\mathrm{ext}}{\dot{q}}^{i}=P_{W}^{\mathrm{ext}},$$

where $P_{W}^{\mathrm{ext}}$ is the power associated with the work done on the system. This is nothing but the statement of the first law for the thermodynamic system, as in Equation (27).

The rate of entropy production of the system is

$$\dot{S}=-\frac{1}{T}\langle F^{\mathrm{fr}},\dot{q}\rangle .$$

The second law states that the internal entropy production is always positive, from which the friction force is dissipative, i.e., $\langle F^{\mathrm{fr}}\left(q,\dot{q},S\right),\dot{q}\rangle \leq 0$ for all $\left(q,\dot{q},S\right)$. This suggests the phenomenological relation $F_{i}^{\mathrm{fr}}=-\lambda_{ij}{\dot{q}}^{j}$, where $\lambda_{ij}$, i,j=1,…,n are functions of the state variables, with the symmetric part of the matrix $\lambda_{ij}$ positive semi-definite, which are determined by experiments.

**Remark** **1** (Phenomenological and variational constraints)**.** *The explicit expression of the constraint in Equation (25) involves phenomenological laws for the friction force $F^{\mathrm{fr}}$, which is why we refer to it as a phenomenological constraint. The associated constraint in Equation (26) is called a variational constraint since it is a condition on the variations to be used in Equation (24). Note that the constraint in Equation (25) is nonlinear and also that one shifts from the variational constraint to the phenomenological constraint by formally replacing the time derivatives $\dot{q}$, $\dot{S}$ by the variations δq, δS:*

$$\frac{\partial L}{\partial S}\dot{S}=\langle F^{\mathrm{fr}},\dot{q}\rangle \;\;⇝\;\;\frac{\partial L}{\partial S}\delta S=\langle F^{\mathrm{fr}},\delta q\rangle .$$

*Such a systematic correspondence between the phenomenological and variational constraints will hold, in general, for our variational formulation of thermodynamics, as we present in detail below.*

**Remark** **2.** *In our macroscopic description, it is assumed that the macroscopically “slow ” or collective motion of the system can be described by q(t), while the time evolution of the entropy S(t) is determined from the microscopically “fast ” motions of molecules through statistical mechanics under the assumption of local equilibrium. It follows from statistical mechanics that the internal energy U(q,S), given as a potential energy at the macroscopic level, is essentially coming from the total kinetic energy associated with the microscopic motion of molecules, which is directly related to the temperature of the system.*

**Figure 1:**
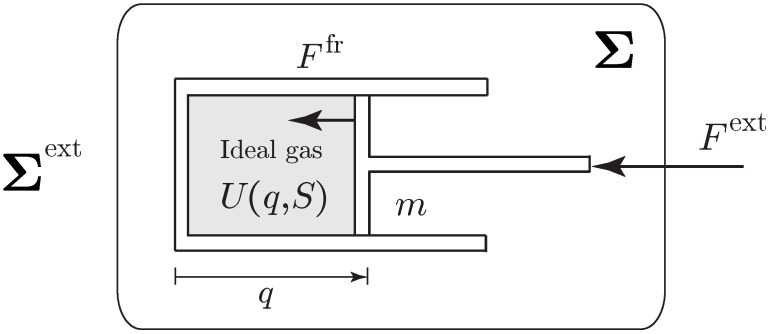
One cylinder.

**Example** **1** (piston)**.** *Consider a gas confined by a piston in a cylinder as in Figure 1. This is an example of a simple adiabatically closed system, whose state can be characterized by (q,v,S).*

*The Lagrangian is given by $L\left(q,v,S\right)=\frac{1}{2}mv^{2}-U\left(q,S\right)$, where m is the mass of the piston; $U\left(q,S\right):=U\left(S,V=Aq,N_{0}\right)$, where U(S,V,N) is the internal energy of the gas, $N_{0}$ is the constant number of moles, V=αq is the volume, and α is the constant area of the cylinder. Note that we have*

$$\frac{\partial U}{\partial S}\left(q,S\right)=T\left(q,S\right)\;and\;\frac{\partial U}{\partial q}\left(q,S\right)=-p\left(q,S\right)\alpha ,$$

*where T is temperature and $p=-\frac{\partial U}{\partial V}$ is the pressure. The friction force reads $F^{\mathrm{fr}}\left(q,\dot{q},S\right)=-\lambda \left(q,S\right)\dot{q}$, where λ(q,S)≥0 is the phenomenological coefficient, which is determined experimentally.*

*Following Equations (24)–(26), the variational formulation is given by*

$$\delta \int_{t_{1}}^{t_{2}}\left[\frac{1}{2}m{\dot{q}}^{2}-U\left(q,S\right)\right]dt+\int_{t_{1}}^{t_{2}}F^{\mathrm{ext}}\left(q,\dot{q},S\right)\delta q\;dt=0,$$

*subject to the phenomenological constraint*

$$\frac{\partial U}{\partial S}\left(q,S\right)\dot{S}=\lambda \left(q,S\right){\dot{q}}^{2}.$$

*and for variations subject to the variational constraint*

$$\frac{\partial U}{\partial S}\left(q,S\right)\delta S=\lambda \left(q,S\right)\dot{q}\delta q.$$

*From this principle, we get the equations of motion for the piston-cylinder system as*

$$m\ddot{q}=p\left(q,S\right)\alpha +F^{\mathrm{ext}}-\lambda \left(q,S\right)\dot{q},\;T\left(q,S\right)\dot{S}=\lambda \left(q,S\right){\dot{q}}^{2},$$

*consistent with the equations derived in Section 4 of [55]. We can verify the energy balance, i.e., the first law, as $\frac{d}{dt}E=F^{\mathrm{ext}}\dot{q}$, where $E=\frac{1}{2}m{\dot{q}}^{2}+U$ is the total energy.*

#### 3.1.2. Variational Formulation for Systems with Internal Mass Transfer

We here extend the previous variational formulation to the finite-dimensional case of discrete systems experiencing internal diffusion processes. Diffusion is particularly important in biology, as many processes depend on the transport of chemical species through bodies. For instance, the setting that we develop is well suited for the description of diffusion across composite membranes, e.g., composed of different elements arranged in a series or parallel array, which occurs frequently in living systems and has remarkable physical properties (see [58,59,60,61]).

**Figure 2:**
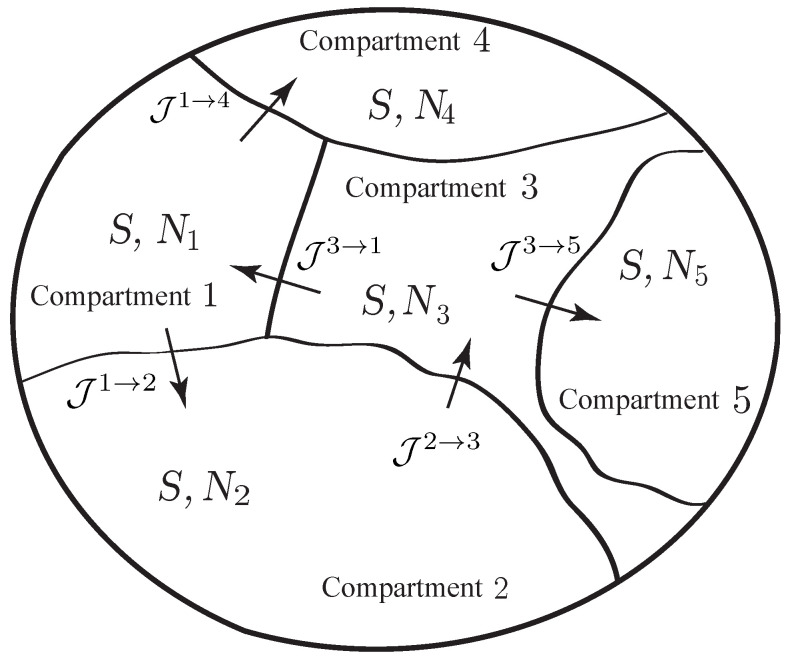
Simple adiabatically closed system with a single chemical species experiencing diffusion among several compartments.

As illustrated in Figure 2, we consider a thermodynamic system consisting of *K* compartments that can exchange matter by diffusion across walls (or membranes) of their common boundaries. We assume that the system has a single species, and we denote by $N_{k}$ the number of moles of the species in the *k*-th compartment, k=1,…,K. We assume that the thermodynamic system is simple; i.e., a uniform entropy *S*, the entropy of the system, is attributed to all the compartments.

For each compartment k=1,…,K, the mole balance equation is

$$\frac{d}{dt}N_{k}=\sum_{\ell =1}^{K}J^{\ell \to k},$$

where $J^{\ell \to k}=-J^{k\to \ell }$ is the molar flow rate from compartment *ℓ* to compartment *k* due to diffusion of the species. We assume that the simple system also involves mechanical variables, friction, and exterior forces $F^{\mathrm{fr}}$ and $F^{\mathrm{ext}}$, as in (A). The Lagrangian of the system is thus a function:

$$L:TQ\times R\times R^{K}\to R,\;\left(q,v,S,N_{1},\ldots ,N_{K}\right)\mapsto L\left(q,v,S,N_{1},\ldots ,N_{K}\right).$$

*Thermodynamic displacements associated with matter exchange.* The variational formulation involves the new variables $W^{k}$, k=1,…,K, which are examples of *thermodynamic displacements* and play a central role in our formulation. In general, we define the *thermodynamic displacement associated with an irreversible process* as the primitive in time of the thermodynamic force (or affinity) of the process. This force (or affinity) thus becomes the rate of change of the thermodynamic displacement. In the case of matter transfer, ${\dot{W}}^{k}$ corresponds to the chemical potential of $N_{k}$.

The variational formulation for a simple system with an internal diffusion process is stated as follows.

Find the curves q(t), S(t), $W^{k}\left(t\right)$, $N_{k}\left(t\right)$ which are critical for the *variational condition*

(29) $$\delta \int_{t_{1}}^{t_{2}}\;\left[L\left(q,\dot{q},S,N_{1},\ldots ,N_{K}\right)+{\dot{W}}^{k}N_{k}\right]dt+\int_{t_{1}}^{t_{2}}\langle \;F^{\mathrm{ext}},\delta q\rangle \;dt=0,$$

subject to the *phenomenological constraint*

(30) $$\frac{\partial L}{\partial S}\dot{S}=\langle F^{\mathrm{fr}},\dot{q}\rangle +\sum_{k,\ell =1}^{K}J^{\ell \to k}{\dot{W}}^{k},$$

and for variations subject to the *variational constraint*

(31) $$\frac{\partial L}{\partial S}\delta S=\langle F^{\mathrm{fr}},\delta q\rangle +\sum_{k,\ell =1}^{K}J^{\ell \to k}\delta W^{k},$$

with $\delta q\left(t_{1}\right)=\delta q\left(t_{2}\right)=0$ and $\delta W^{k}\left(t_{1}\right)=\delta W^{k}\left(t_{2}\right)=0$, k=1,…,K.

Taking variations of the integral in Equation (29), integrating by parts, and using $\delta q\left(t_{1}\right)=\delta q\left(t_{2}\right)=0$ and $\delta W^{k}\left(t_{1}\right)=\delta W^{k}\left(t_{2}\right)=0$, it follows that

$$\int_{t_{1}}^{t_{2}}\left[\left(\frac{\partial L}{\partial q^{i}}-\frac{d}{dt}\frac{\partial L}{\partial {\dot{q}}^{i}}+F_{i}^{\mathrm{ext}}\right)\delta q^{i}+\frac{\partial L}{\partial S}\delta S+\left(\frac{\partial L}{\partial N_{k}}+{\dot{W}}^{k}\right)\delta N_{k}-{\dot{N}}_{k}\delta W^{k}\right]\;dt.$$

Then, using the variational constraint in Equation (31), we get the following conditions:

(32) $$\begin{array}{cc} \delta q^{i}: & \;\frac{d}{dt}\frac{\partial L}{\partial {\dot{q}}^{i}}-\frac{\partial L}{\partial q^{i}}=F_{i}^{\mathrm{fr}}+F_{i}^{\mathrm{ext}},\;i=1,\ldots ,n,\\ \delta N_{k}: & \;\frac{d}{dt}W^{k}=-\frac{\partial L}{\partial N_{k}},\;k=1,\ldots ,K,\\ \delta W^{k}: & \;\frac{d}{dt}N_{k}=\sum_{\ell =1}^{K}J^{\ell \to k},\;k=1,\ldots ,K. \end{array}$$

These conditions, combined with the phenomenological constraint in Equation (30), yield the system of evolution equations for the curves q(t), S(t), and $N^{k}\left(t\right)$:

(33) $$\left\{\begin{array}{c} \;\frac{d}{dt}\frac{\partial L}{\partial \dot{q}}-\frac{\partial L}{\partial q}=F^{\mathrm{fr}}+F^{\mathrm{ext}},\\ \;\frac{d}{dt}N_{k}=\sum_{\ell =1}^{K}J^{\ell \to k},\;k=1,\ldots ,K,\\ \frac{\partial L}{\partial S}\dot{S}=\langle F^{\mathrm{fr}},\dot{q}\rangle -\sum_{k<\ell }J^{\ell \to k}\left(\frac{\partial L}{\partial N_{k}}-\frac{\partial L}{\partial N_{\ell }}\right). \end{array}\right.$$

The total energy is defined as in Equations (5) and (28) and depends here on the mechanical variables (q,v)∈TQ, the entropy *S*, and the number of moles $N_{k}$, k=1,…,K, i.e., we define $E:TQ\times R\times R^{K}\to R$ as

(34) $$E\left(q,v,S,N_{1},\ldots ,N_{K}\right)=\langle \frac{\partial L}{\partial v},v\rangle -L\left(q,v,S,N_{1},\ldots ,N_{K}\right).$$

On the solutions of Equation (33), we have

$$\frac{d}{dt}E=\left(\frac{d}{dt}\frac{\partial L}{\partial {\dot{q}}^{i}}-\frac{\partial L}{\partial q^{i}}\right){\dot{q}}^{i}-\frac{\partial L}{\partial S}\dot{S}-\frac{\partial L}{\partial N_{k}}{\dot{N}}_{k}=F_{i}^{\mathrm{ext}}{\dot{q}}^{i}=P_{W}^{\mathrm{ext}},$$

where $P_{W}^{\mathrm{ext}}$ is the power associated with the work done on the system. This is the statement of the first law for the thermodynamic system in Equation (33).

For a given Lagrangian *L*, the temperature and chemical potentials of each compartment are defined as

$$T:=-\frac{\partial L}{\partial S}\;\mathrm{and}\;\mu^{k}:=-\frac{\partial L}{\partial N_{k}},\;\;k=1,\ldots ,K.$$

The last equation in Equation (33) yields the rate of entropy production of the system as

$$\dot{S}=-\frac{1}{T}\langle F^{\mathrm{fr}},\dot{q}\rangle +\frac{1}{T}\sum_{k<\ell }J^{k\to \ell }\left(\mu^{k}-\mu^{\ell }\right),$$

where the two terms correspond, respectively, to the rate of entropy production due to mechanical friction and to matter transfer. The second law suggests the phenomenological relations

$$F_{i}^{\mathrm{fr}}=-\lambda_{ij}{\dot{q}}^{j}\;\mathrm{and}\;J^{k\to \ell }=G^{kl}\left(\mu^{k}-\mu^{\ell }\right),$$

where $\lambda_{ij}$, i,j=1,…,n and $G^{k\ell }$, k,ℓ=1,…,K are functions of the state variables, with the symmetric part of the matrix $\lambda_{ij}$ positive semi-definite and with $G^{k\ell }\geq 0$ for all k,ℓ.

**Figure 3:**
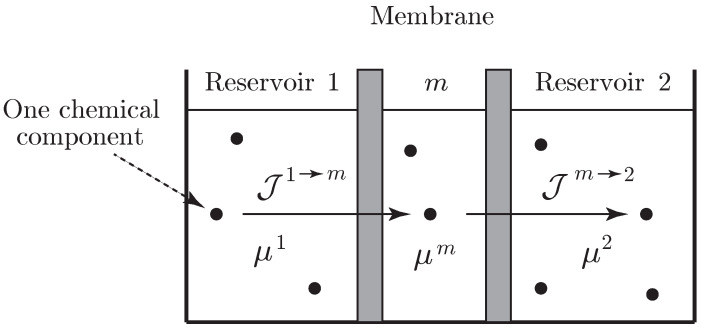
Nonelectrolyte diffusion through a homogeneous membrane.

**Example** **2** (mass transfer associated with nonelectrolyte diffusion through a homogeneous membrane)**.** *We consider a system with diffusion due to internal matter transfer through a homogeneous membrane separating two reservoirs. We suppose that the system is simple (so it is described by a single entropy variable) and involves a single chemical species. We assume that the membrane consists of three regions, namely, the central layer denotes the membrane capacitance in which energy is stored without dissipation, while the outer layers indicate transition regions in which dissipation occurs with no energy storage. We denote by $N_{m}$ the number of mole of this chemical species in the membrane and by $N_{1}$ and $N_{2}$ the numbers of mole in reservoirs 1 and 2, as shown in Figure 3. Define the Lagrangian by $L\left(S,N_{1},N_{2},N_{m}\right)=-U\left(S,N_{1},N_{2},N_{m}\right)$, where $U(S,N_{1},N_{2},N_{m})$ denotes the internal energy of the system, and assume that the volumes are constant and the system is isolated. We denote by $\mu^{k}=\frac{\partial U}{\partial N_{k}}$ the chemical potential of the chemical species in the reservoirs (k=1,2) and in the membrane (k=m). The flux from reservoir 1 into the membrane is denoted by $J^{1\to m}$, and the flux from the membrane into reservoir 2 is denoted by $J^{m\to 2}$.*

*The variational condition for the diffusion process is provided by*

(35) $$\delta \int_{t_{1}}^{t_{2}}\left[L\left(S,N_{1},N_{2},N_{m}\right)+{\dot{W}}^{1}N_{1}+{\dot{W}}^{2}N_{2}+{\dot{W}}^{m}N_{m}\right]dt=0,$$

*subject to the phenomenological constraint*

(36) $$\frac{\partial L}{\partial S}\dot{S}=J^{m\to 1}\left({\dot{W}}^{1}-{\dot{W}}^{m}\right)+J^{m\to 2}\left({\dot{W}}^{2}-{\dot{W}}^{m}\right)$$

*and for variations subject to the variational constraint*

(37) $$\frac{\partial L}{\partial S}\delta S=J^{m\to 1}(\delta W^{1}-\delta W^{m)}+J^{m\to 2}\left(\delta W^{2}-\delta W^{m}\right),$$

*with $\delta W^{k}\left(t_{i}\right)=0$ for k=1,2,m and i=1,2.*

*Thus, it follows that*

(38) $${\dot{N}}_{1}=J^{m\to 1},\;{\dot{N}}_{m}=J^{1\to m}+J^{2\to m},\;{\dot{N}}_{2}=J^{m\to 2}$$

*and ${\dot{W}}^{1}=\mu^{1},\;{\dot{W}}^{2}=\mu^{2},\;{\dot{W}}^{m}=\mu^{m}$. The constraint in Equation (36) becomes*

(39) $$-T\dot{S}=J^{m\to 1}\left(\mu^{1}-\mu^{m}\right)+J^{m\to 2}\left(\mu^{2}-\mu^{m}\right),$$

*where $T=-\frac{\partial L}{\partial S}$. Equations (38) and (39) are equivalent to those derived in ([61] Section 2.2). From Equations (38) and (39), we have energy conservation $\frac{d}{dt}U=0$, which is consistent with the fact that the system is isolated.*

### 3.2. Adiabatically Closed Non-Simple Thermodynamic Systems

**Figure 4:**
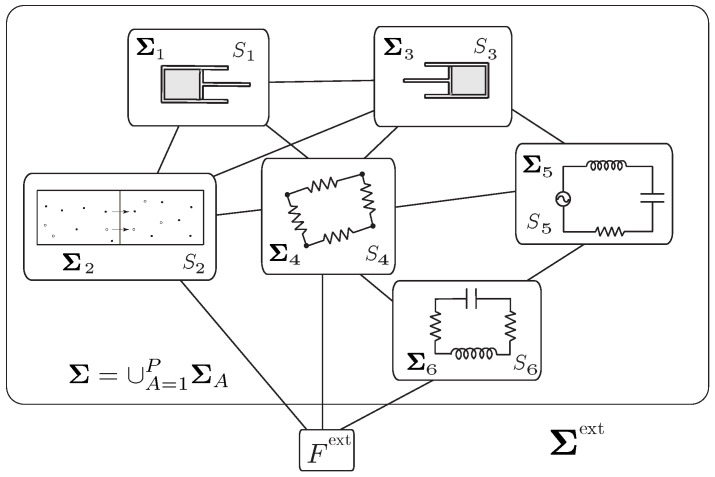
Non-simple interconnected system.

We now consider a general finite-dimensional system $\Sigma =\cup_{A=1}^{P}\Sigma_{A}$ composed of interconnected simple thermodynamic systems $\Sigma_{A}$, as illustrated in Figure 4. This class of non-simple interconnected systems extends the class of *interconnected mechanical systems* (see [62]) to include the irreversible processes. In addition to the irreversible processes of friction and mass transfer described earlier, these systems can also involve the process of heat conduction.

The main difference from the previous cases is the occurrence of several entropy variables, namely, each subsystem $\Sigma_{A}$ has an entropy denoted by $S_{A},\;A=1,\ldots ,P$. Besides the variables $S_{A}$, each subsystem $\Sigma_{A}$ may also be described by mechanical variables $q^{A}\in Q_{A}$ and number of moles $\left(N_{A,1},\ldots ,N_{A,K_{A}}\right)\in R^{K_{A}}$, where $Q_{A}$ is a configuration manifold for a mechanical variable associated with $\Sigma_{A}$ and where $K_{A}$ is the number of compartments in a simple system $\Sigma_{A}$. For simplicity, we assume that independent mechanical coordinates q∈Q have been chosen to represent the mechanical configuration of the interconnected system Σ. The state variables needed to describe this system are

(40) $$\left(q,v\right)\in TQ,\;S_{A},\;A=1,\ldots ,P,\;N_{A,k},\;k=1,\ldots ,K_{A},\;A=1,\ldots ,P.$$

We present the variational formulation for these systems in two steps, exactly as in Section 3.1, by first considering the case without any transfer of mass.

#### 3.2.1. Variational Formulation for Systems with Friction and Heat Conduction

Besides the entropies $S_{A}$, A=1,…,P, these systems only involve mechanical variables. The Lagrangian of the system is thus a function:

$$L:TQ\;\times R^{P}\to R,\;\left(q,v,S_{1},\ldots ,S_{P}\right)\mapsto L\left(q,v,S_{1},\ldots ,S_{P}\right).$$

We denote by $F^{\mathrm{ext}\to A}:T^{*}Q\times R^{P}\to T^{*}Q$ the external force acting on subsystem $\Sigma_{A}$. Consistent with the fact that the mechanical variables $q=(q^{1},\ldots ,q^{n})$ describe the configuration of the entire interconnected system Σ, only the total exterior force $F^{\mathrm{ext}}=\sum_{A=1}^{P}F^{\mathrm{ext}\to A}$ appears explicitly in the variational condition in Equation (42). We denote by $F^{\mathrm{fr}(A)}:T^{*}Q\times R^{P}\to T^{*}Q$ the friction forces experienced by subsystem $\Sigma_{A}$. This friction force is at the origin of an entropy production for subsystem $\Sigma_{A}$ and appears explicitly in the phenomenological constraint (Equation (43)) and the variational constraint (Equation (44)) of the variational formulation. We also introduce the fluxes $J_{AB}$, A≠B associated with the heat exchange between subsystems $\Sigma_{A}$ and $\Sigma_{B}$ and such that $J_{AB}=J_{BA}$. The relation between the fluxes $J_{AB}$ and the heat power exchange $P_{H}^{A\to B}$ are given later. For the construction of variational structures, it is convenient to define the flux $J_{AB}$ for A=B as

$$J_{AA}:=-\sum_{B\neq A}J_{AB},$$

so that we have

(41) $$\sum_{A=1}^{P}J_{AB}=0,\;\mathrm{for}\;\mathrm{all}\;B.$$

*Thermodynamic displacements associated with heat exchange.* To incorporate heat exchange into our variational formulation, the new variables $\Gamma^{A}$, A=1,…,P are introduced. These are again examples of *thermodynamic displacements* in the same way as we defined $W^{k}$ before. For the case of heat exchange, ${\dot{\Gamma }}^{A}$ corresponds to the temperature of the subsystem $\Sigma_{A}$, where $\Gamma^{A}$ is identical to the *thermal displacement* employed in [37], which was originally introduced by [63]. The introduction of $\Gamma^{A}$ is accompanied by the introduction of an entropy variable $\Sigma_{A}$ whose meaning will be clarified later.

Now, the variational formulation for a system with friction and heat conduction is stated as follows:

Find the curves q(t), $S_{A}\left(t\right)$, $\Gamma^{A}\left(t\right)$, $\Sigma_{A}\left(t\right)$ which are critical for the *variational condition*

(42) $$\delta \int_{t_{1}}^{t_{2}}\;\left[L\left(q,\dot{q},S_{1},\ldots ,S_{K}\right)+{\dot{\Gamma }}^{A}\left(S_{A}-\Sigma_{A}\right)\right]dt+\int_{t_{1}}^{t_{2}}\langle F^{\mathrm{ext}},\delta q\rangle \;dt=0,$$

subject to the *phenomenological constraint*

(43) $$\frac{\partial L}{\partial S_{A}}{\dot{\Sigma }}_{A}=\langle F^{\mathrm{fr}(A)},\dot{q}\rangle +J_{AB}{\dot{\Gamma }}^{B},\;\mathrm{for}\;A=1,\ldots ,P,$$

and for variations subject to the *variational constraint*

(44) $$\frac{\partial L}{\partial S_{A}}\delta \Sigma_{A}=\langle F^{\mathrm{fr}(A)},\delta q\rangle +J_{AB}\delta \Gamma^{B},\;\mathrm{for}\;A=1,\ldots ,P,$$

with $\delta q\left(t_{1}\right)=\delta q\left(t_{2}\right)=0$ and $\delta \Gamma^{A}\left(t_{1}\right)=\delta \Gamma^{A}\left(t_{2}\right)=0$, A=1,…,P.

Taking variations of the integral in Equation (42), integrating by parts, and using $\delta q\left(t_{1}\right)=\delta \left(t_{2}\right)=0$ and $\delta \Gamma_{A}\left(t_{1}\right)=\delta \Gamma_{A}\left(t_{2}\right)=0$, it follows that

$$\int_{t_{1}}^{t_{2}}\left[\left(\frac{\partial L}{\partial q^{i}}-\frac{d}{dt}\frac{\partial L}{\partial {\dot{q}}^{i}}+F_{i}^{\mathrm{ext}}\right)\delta q^{i}+\frac{\partial L}{\partial S_{A}}\delta S_{A}-\left({\dot{S}}_{A}-{\dot{\Sigma }}_{A}\right)\delta \Gamma^{A}+{\dot{\Gamma }}^{A}\left(\delta S_{A}-\delta \Sigma_{A}\right)\right]dt=0.$$

Then, using the variational constraint (Equation (44)), we get the following conditions:

$$\begin{array}{cc} \delta q^{i}: & \;\frac{\partial L}{\partial q^{i}}-\frac{d}{dt}\frac{\partial L}{\partial {\dot{q}}^{i}}-\sum_{A=1}^{P}\frac{{\dot{\Gamma }}^{A}}{\frac{\partial L}{\partial S_{A}}}F_{i}^{\mathrm{fr}(A)}+F_{i}^{\mathrm{ext}}=0,\;i=1,\ldots ,n,\\ \delta S_{A}: & \;\frac{\partial L}{\partial S_{A}}+{\dot{\Gamma }}^{A}=0,\;A=1,\ldots ,P,\\ \delta \Gamma^{A}: & \;-{\dot{S}}_{A}+{\dot{\Sigma }}_{A}-\sum_{B=1}^{P}\frac{{\dot{\Gamma }}^{A}}{\frac{\partial L}{\partial S_{A}}}J_{BA}=0,\;A=1,\ldots ,P. \end{array}$$

The second equation yields

(45) $${\dot{\Gamma }}^{A}=-\frac{\partial L}{\partial S_{A}}=:T^{A},$$

where $T^{A}$ is the temperature of the subsystem $\Sigma_{A}$. This implies that $\Gamma_{A}$ is a thermal displacement. Because of Equation (41), the last equation yields ${\dot{S}}_{A}={\dot{\Sigma }}_{A}$. Hence, using Equation (43), we get the following system of evolution equations for the curves q(t) and $S_{A}\left(t\right)$:

(46) $$\left\{\begin{array}{c} \;\frac{d}{dt}\frac{\partial L}{\partial \dot{q}}-\frac{\partial L}{\partial q}=\sum_{A=1}^{P}F^{\mathrm{fr}(A)}+F^{\mathrm{ext}},\\ \frac{\partial L}{\partial S_{A}}{\dot{S}}_{A}=\langle F^{\mathrm{fr}(A)},\dot{q}\rangle -\sum_{B=1}^{P}J_{AB}\left(\frac{\partial L}{\partial S_{B}}-\frac{\partial L}{\partial S_{A}}\right),\;A=1,\ldots ,P. \end{array}\right.$$

As before, we have $\frac{d}{dt}E=\langle F^{\mathrm{ext}},\dot{q}\rangle =P_{W}^{\mathrm{ext}}$, where the total energy *E* is defined in the same way as before. Since the system is *non-simple*, it is instructive to analyze the energy behavior of each subsystem. This can be done if the Lagrangian is given by the sum of the Lagrangians of the subsystems, i.e.,

$$L\left(q,v,S_{1},\ldots ,S_{P}\right)=\sum_{A=1}^{P}L_{A}\left(q,v,S_{A}\right).$$

The mechanical equation for $\Sigma_{A}$ is given as

$$\frac{d}{dt}\frac{\partial L_{A}}{\partial \dot{q}}-\frac{\partial L_{A}}{\partial q}=F^{\mathrm{fr}(A)}+F^{\mathrm{ext}\to A}+\sum_{B=1}^{P}F^{B\to A},$$

where $F^{B\to A}=-F^{A\to B}$ is the internal force exerted by $\Sigma_{B}$ on $\Sigma_{A}$. Denoting $E_{A}$ as the total energy of $\Sigma_{A}$, we have

(47) $$\begin{array}{cc} \frac{d}{dt}E_{A} & =\langle F^{\mathrm{ext}\to A},\dot{q}\rangle +\sum_{B=1}^{P}\langle F^{B\to A},\dot{q}\rangle +\sum_{B=1}^{P}J_{AB}\left(\frac{\partial L}{\partial S_{B}}-\frac{\partial L}{\partial S_{A}}\right)\\ & =P_{W}^{\mathrm{ext}\to A}+\sum_{B=1}^{P}P_{W}^{B\to A}+\sum_{B=1}^{P}P_{H}^{B\to A}, \end{array}$$

where $P_{W}^{\mathrm{ext}\to A}$ and $P_{W}^{B\to A}$ denote the power associated with the work done on $\Sigma_{A}$ by the exterior and that by the subsystem $\Sigma_{B}$, respectively, and where $P_{H}^{B\to A}$ is the power associated with the heat transfer from $\Sigma_{B}$ to $\Sigma_{A}$. The link between the flux $J_{AB}$ and the power exchange is thus

$$P_{H}^{B\to A}=J_{AB}\left(T^{A}-T^{B}\right).$$

Since entropy is an extensive variable, the total entropy of the system is $S=\sum_{A=1}^{P}S_{A}$. From Equation (46), it follows that the rate of total entropy production $\dot{S}=\sum_{A=1}^{P}{\dot{S}}_{A}$ of the system is given by

(48) $$\dot{S}=-\sum_{A=1}^{P}\frac{1}{T^{A}}\langle F^{\mathrm{fr}(A)},\dot{q}\rangle +\sum_{A<B}^{K}J_{AB}\left(\frac{1}{T^{B}}-\frac{1}{T^{A}}\right)\left(T^{B}-T^{A}\right).$$

The second law suggests the phenomenological relations

(49) $$F_{i}^{\mathrm{fr}(A)}=-\lambda_{ij}^{A}{\dot{q}}^{j}\;\mathrm{and}\;J_{AB}\frac{T^{A}-T^{B}}{T^{A}T^{B}}=L_{AB}\left(T^{B}-T^{A}\right),$$

where $\lambda_{ij}^{A}$ and $L_{AB}$ are functions of the state variables, with the symmetric part of the matrices $\lambda_{ij}^{A}$ positive semi-definite and with $L_{AB}\geq 0$ for all A,B. From the second relation, we deduce $J_{AB}=-L_{AB}T^{A}T^{B}=-\kappa_{AB}$, with $\kappa_{AB}=\kappa_{AB}\left(q,S_{A},S_{B}\right)$ being the heat conduction coefficients between subsystem $\Sigma_{A}$ and subsystem $\Sigma_{B}$.

**Figure 5:**
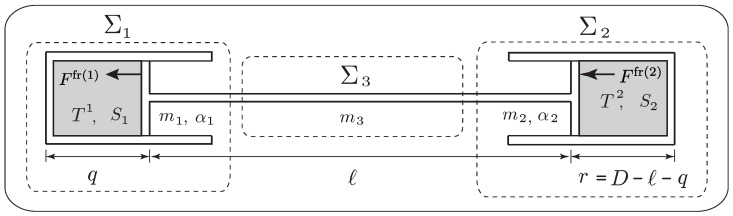
The two-cylinder problem.

**Example** **3** (The adiabatic piston)**.** *We consider a piston-cylinder system composed of two cylinders connected by a rod, each of which contains a fluid (or an ideal gas) and is separated by a movable piston, as shown in Figure 5. We assume that the system is isolated. Despite its apparent simplicity, this system has attracted a lot of attention in the literature because there has been some controversy about the final equilibrium state of this system when the piston is adiabatic. We refer to [55] for a review of this challenging problem and for the derivation of the time evolution of this system, based on the approach of [38].*

*The system ***Σ*** may be regarded as an interconnected system consisting of three simple systems; namely, the two pistons $\Sigma_{1},\Sigma_{2}$ of mass $m_{1},m_{2}$ and the connecting rod $\Sigma_{3}$ of mass $m_{3}$. As illustrated in Figure 5, q and r=D−ℓ−q denote, respectively, the distance between the bottom of each piston to the top, where D is a constant. In this setting, we choose the variables $\left(q,v,S_{1},S_{2}\right)$ (the entropy associated with $\Sigma_{3}$ is constant) to describe the dynamics of the interconnected system, and the Lagrangian is given by*

(50) $$L\left(q,v,S_{1},S_{2}\right)=\frac{1}{2}Mv^{2}-U_{1}\left(q,S_{1}\right)-U_{2}\left(q,S_{2}\right),$$

*where $M:=m_{1}+m_{2}+m_{3}$, and*

$$U_{1}\left(q,S_{1}\right):=U_{1}\left(S_{1},V_{1}=\alpha_{1}q,N_{1}\right),\;U_{2}\left(q,S_{2}\right):=U_{2}\left(S_{2},V_{2}=\alpha_{2}r,N_{2}\right),$$

*with $U_{i}\left(S_{i},V_{i},N_{i}\right)$ as the internal energies of the fluids, $N_{i}$ as the constant number of moles, and $\alpha_{i}$ as the constant areas of the cylinders, i=1,2.*

*As in Equation (49), we have $F^{\mathrm{fr}(A)}\left(q,\dot{q},S_{A}\right)=-\lambda^{A}\dot{q}$, with $\lambda^{A}=\lambda^{A}\left(q,S^{A}\right)\geq 0$, A=1,2 and $J_{AB}=-\kappa_{AB}=:-\kappa$, where $\kappa =\kappa (S_{1},S_{2},q)\geq 0$ is the heat conductivity of the connecting rod.*

*From the variational formulations (Equations (42)–(44)), we get the following system for q(t), $S_{1}\left(t\right)$, $S_{2}\left(t\right)$, in light of Equation (46), as*

$$\left\{\begin{array}{c} \;M\ddot{q}=p_{1}\left(q,S_{1}\right)\alpha_{1}-p_{2}\left(q,S_{2}\right)\alpha_{2}-\left(\lambda^{1}+\lambda^{2}\right)\dot{q},\\ \;T^{1}\left(q,S_{1}\right){\dot{S}}_{1}=\lambda^{1}{\dot{q}}^{2}+\kappa \left(T^{2}\left(q,S_{2}\right)-T^{1}\left(q,S_{1}\right)\right),\\ T^{2}\left(q,S_{2}\right){\dot{S}}_{2}=\lambda^{2}{\dot{q}}^{2}+\kappa \left(T^{1}\left(q,S_{1}\right)-T^{2}\left(q,S_{2}\right)\right), \end{array}\right.$$

*where we used $\frac{\partial U_{i}}{\partial S_{i}}\left(q,S_{i}\right)=T^{i}\left(q,S_{i}\right)$, $\frac{\partial U_{1}}{\partial q}=-p_{1}\left(q,S_{1}\right)\alpha_{1}$, and $\frac{\partial U_{2}}{\partial q}=p_{2}\left(q,S_{2}\right)\alpha_{2}$.*

*These equations recover those derived in [55], (51)–(53). We have $\frac{d}{dt}E=0$, where $E=\frac{1}{2}M{\dot{q}}^{2}+U_{1}\left(q,S_{1}\right)+U\left(q,S_{2}\right)$, consistent with the fact that the system is isolated. The rate of total entropy production is*

$$\frac{d}{dt}S=\left(\frac{\lambda^{1}}{T^{1}}+\frac{\lambda^{2}}{T^{2}}\right){\dot{q}}^{2}+\kappa \frac{{\left(T^{2}-T^{1}\right)}^{2}}{T^{1}T^{2}}\geq 0.$$

*The equations of motion for the adiabatic piston are obtained by setting κ=0.*

#### 3.2.2. Variational Formulation for Systems with Friction, Heat Conduction, and Internal Mass Transfer

We extend the previous case to one in which the subsystems $\Sigma_{A}$ not only exchange work and heat but also exchange matter. In general, each subsystem may itself have several compartments, in which case the variables are those listed in Equation (40). For simplicity, we assume that each subsystem has only one compartment. The reader can easily extend this approach to the general case. The Lagrangian is thus a function:

$$L:TQ\times R^{P}\times R^{P}\to R,\;\left(q,v,S_{1},\ldots ,S_{P},N_{1},\ldots ,N_{P}\right)\mapsto L\left(q,v,S_{1},\ldots ,S_{P},N_{1},\ldots ,N_{P}\right),$$

where $S_{A}$ and $N_{A}$ are the entropy and number of moles of subsystem $\Sigma_{A}$, A=1,…,P. Since the previous cases are presented in detail above, we here just present the variational formulation and the resulting equations of motion.

Find the curves q(t), $S_{A}\left(t\right)$, $\Gamma^{A}\left(t\right)$, $\Sigma_{A}\left(t\right)$, $W^{A}\left(t\right)$, $N_{A}\left(t\right)$ which are critical for the *variational condition*

(51) $$\begin{array}{c} \delta \int_{t_{1}}^{t_{2}}\;\left[L\left(q,\dot{q},S_{1},\ldots ,S_{P},N_{1},\ldots ,N_{P}\right)+{\dot{W}}^{A}N_{A}+{\dot{\Gamma }}^{A}\left(S_{A}-\Sigma_{A}\right)\right]dt+\int_{t_{1}}^{t_{2}}\langle F^{\mathrm{ext}},\delta q\rangle dt=0, \end{array}$$

subject to the *phenomenological constraint*

(52) $$\frac{\partial L}{\partial S_{A}}{\dot{\Sigma }}_{A}=\langle F^{\mathrm{fr}(A)},\dot{q}\rangle +J_{AB}{\dot{\Gamma }}^{B}+J^{B\to A}{\dot{W}}^{A},\;\mathrm{for}\;A=1,\ldots ,P,$$

and for variations subject to the *variational constraint*

(53) $$\frac{\partial L}{\partial S_{A}}\delta \Sigma_{A}=\langle F^{\mathrm{fr}(A)},\;\delta q\rangle +J_{AB}\delta \Gamma^{B}+J^{B\to A}\delta W^{A},\;\mathrm{for}\;A=1,\ldots ,P,$$

with $\delta q\left(t_{i}\right)=\delta W^{A}\left(t_{i}\right)=\delta \Gamma^{A}\left(t_{i}\right)=0$, i=1,2, A=1,…,P.

From Equations (51)–(53), we obtain the following system of evolution equations for the curves q(t), $S_{A}\left(t\right)$, and $N_{A}\left(t\right)$:

(54) $$\left\{\begin{array}{c} \;\frac{d}{dt}\frac{\partial L}{\partial \dot{q}}-\frac{\partial L}{\partial q}=\sum_{A=1}^{P}F^{\mathrm{fr}(A)}+F^{\mathrm{ext}},\\ \;\frac{d}{dt}N_{A}=\sum_{B=1}^{P}J^{B\to A},\;A=1,\ldots ,P,\\ \;\frac{\partial L}{\partial S_{A}}{\dot{S}}_{A}=\langle F^{\mathrm{fr}(A)},\dot{q}\rangle -\sum_{B=1}^{P}J_{AB}\left(\frac{\partial L}{\partial S_{B}}-\frac{\partial L}{\partial S_{A}}\right)-\sum_{B=1}^{P}J^{B\to A}\frac{\partial L}{\partial N_{A}},\;A=1,\ldots ,P. \end{array}\right.$$

We also obtain the conditions

$${\dot{\Gamma }}^{A}=-\frac{\partial L}{\partial S_{A}}=:T^{A},\;{\dot{W}}^{A}=-\frac{\partial L}{\partial N_{A}}=:\mu^{A},\;{\dot{\Sigma }}_{A}={\dot{S}}_{A},\;A=1,\ldots ,P,$$

where we defined the temperature $T^{A}$ and the chemical potential $\mu^{A}$ of the subsystem $\Sigma_{A}$. The variables $\Gamma^{A}$ and $W^{A}$ are again the thermodynamic displacements associated with the processes of heat and matter transfer.

The total energy satisfies $\frac{d}{dt}E=P_{W}^{\mathrm{ext}}$ and the detailed energy balances can be carried out as in Equation (47) and yields here

$$P_{H+M}^{B\to A}=J_{AB}\left(T^{A}-T^{B}\right).$$

The rate of total entropy production of the system is computed as

$$\dot{S}=-\sum_{A=1}^{P}\frac{1}{T^{A}}\langle F^{\mathrm{fr}(A)},\dot{q}\rangle +\sum_{A<B}J_{AB}\left(\frac{1}{T^{B}}-\frac{1}{T^{A}}\right)\left(T^{B}-T^{A}\right)+\sum_{A<B}J^{B\to A}\left(\frac{\mu^{B}}{T^{B}}-\frac{\mu^{A}}{T^{A}}\right).$$

From the second law of thermodynamics, the total entropy production must be positive and hence suggests the phenomenological relations

(55) $$F_{i}^{\mathrm{fr}(A)}=-\lambda_{ij}^{A}{\dot{q}}^{j},\;\;\left[\begin{array}{c} \;\frac{T^{A}-T^{B}}{T^{A}T^{B}}J_{AB}\\ J^{B\to A} \end{array}\right]=L_{AB}\left[\begin{array}{c} \;T^{B}-T^{A}\\ \frac{\mu^{B}}{T^{B}}-\frac{\mu^{A}}{T^{A}} \end{array}\right],$$

where the symmetric part of the n×n matrices $\lambda^{A}$ and of the 2×2 matrices $L_{AB}$ are positive. The entries of these matrices are phenomenological coefficients determined experimentally, which may generally depend on the state variables. From Onsager’s reciprocal relations, the 2×2 matrix

$$L_{AB}=\left[\begin{array}{cc} \;L_{AB}^{HH} & L_{AB}^{HM}\\ L_{AB}^{MH} & L_{AB}^{MM} \end{array}\right]$$

is symmetric for all A,B. The matrix elements $L_{AB}^{HH}$ and $L_{AB}^{MM}$ are related to the processes of heat conduction and diffusion between $\Sigma_{A}$ and $\Sigma_{B}$. The coefficients $L_{AB}^{MH}$ and $L_{AB}^{HM}$ describe the cross-effects, and hence are associated with discrete versions of the process of thermal diffusion and the Dufour effect. Thermal diffusion is the process of matter diffusion due to the temperature difference between the compartments. The Dufour effect is the process of heat transfer due to difference of chemical potentials between the compartments.

**Figure 6:**
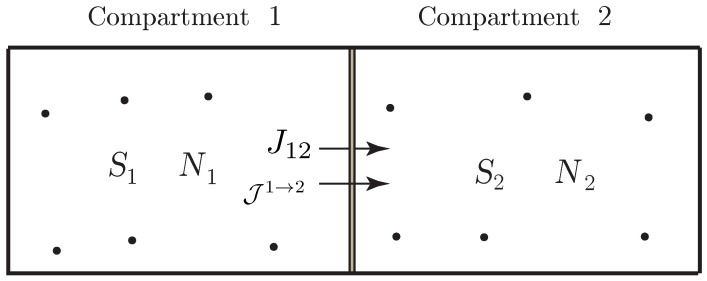
Non-simple closed system with a single chemical species, experiencing diffusion and heat conduction between two compartments.

**Example** **4** (Heat conduction and diffusion between two compartments)**.** *We consider a closed system consisting of two compartments, as illustrated in Figure 6. The compartments are separated by a permeable wall through which heat conduction and diffusion is possible. The system is closed and, therefore, there is no matter transfer with exterior, while we have heat and mass transfer between the compartments.*

*The Lagrangian of this system is*

$$L\left(S_{1},S_{2},N_{1},N_{2}\right)=-U_{1}\left(S_{1},N_{1}\right)-U_{2}\left(S_{2},N_{2}\right),$$

*where $U_{i}\left(S_{i},N_{i}\right)$ is the internal energy of the ith chemical species and the volume is assumed to be constant. In this case, the system in Equation (54) specifies*

(56) $$\left\{\begin{array}{c} {\dot{N}}_{1}=J^{2\to 1},\;{\dot{N}}_{2}=J^{1\to 2},\\ T^{1}{\dot{S}}_{1}=-J^{12}\left(T^{2}-T^{1}\right)-J^{2\to 1}\mu^{1},\\ T^{2}{\dot{S}}_{2}=-J^{12}\left(T^{1}-T^{2}\right)-J^{1\to 2}\mu^{2}, \end{array}\right.$$

*where*

$$T^{A}=\frac{\partial U}{\partial S_{A}},\;\mu^{A}=\frac{\partial U}{\partial N_{A}},\;\;A=1,2$$

*are the temperatures and chemical potentials of the Ath compartments. From Equation (56), it follows that the equation for the total entropy $S=S_{1}+S_{2}$ of the system is*

$$\dot{S}=J^{12}\left(T^{1}-T^{2}\right)\left(\frac{1}{T^{1}}-\frac{1}{T^{2}}\right)+J^{1\to 2}\left(\frac{\mu^{1}}{T^{1}}-\frac{\mu^{2}}{T^{2}}\right)\geq 0,$$

*from which the phenomenological relations are obtained as in Equation (55). The energy balance in each compartment is*

$$\frac{d}{dt}U_{1}=-J^{12}\left(T^{2}-T^{1}\right),\;\frac{d}{dt}U_{2}=-J^{12}\left(T^{1}-T^{2}\right),$$

*which shows the relation between the flux $J^{12}$ and the power $P^{1\to 2}=J^{12}\left(T^{2}-T^{1}\right)$ exchanged between the two compartments due to heat conduction, diffusion, and their cross-effects. The total energy $E=U_{1}+U_{2}$ is conserved.*

**Remark** **3** (General structure of the variational formulation for adiabatically closed systems)**.** *In each of the situation considered, the variational constraint can be systematically obtained from the phenomenological constraint by replacing the time derivative by the delta variation for each process. For the most general case treated above, we have the following correspondence:*

$$\begin{array}{c} \frac{\partial L}{\partial S_{A}}{\dot{\Sigma }}_{A}=\langle F^{\mathrm{fr}(A)},\dot{q}\rangle +J_{AB}{\dot{\Gamma }}^{B}+J^{B\to A}{\dot{W}}^{A}\;⇝\;\frac{\partial L}{\partial S_{A}}\delta \Sigma_{A}=\langle F^{\mathrm{fr}(A)},\delta q\rangle +J_{AB}\delta \Gamma^{B}+J^{B\to A}\delta W^{A}. \end{array}$$

*In the above, the quantities to be determined from the state variables by phenomenological laws are $F^{\mathrm{fr}(A)}$, $J_{AB}$, and $J^{B\to A}$.*

*The structure of our variational formulation is better explained by adopting a general point of view. If we denote by Q the thermodynamic configuration manifold and by x∈Q the collection of all the variables of the thermodynamic system, for instance, $x=(q,S_{A},N_{A},W^{A},\Gamma^{A},\Sigma_{A})$, A=1,…,P in the preceding case, then the variational formulation for an adiabatically closed system falls into the following abstract setting. Given a Lagrangian L:TQ→R, an external force $F^{\mathrm{ext}}:TQ\to T^{*}Q$, and fiber-preserving maps $A^{\alpha }:TQ\to T^{*}Q$, $A^{\alpha }\left(x,v\right)\in T_{x}^{*}Q$, α=1,…,k, the variational formulation reads as follows:*

(57) $$\delta \int_{t_{1}}^{t_{2}}L\left(x\left(t\right),\dot{x}\left(t\right)\right)dt+\int_{t_{1}}^{t_{2}}\langle F^{\mathrm{ext}}\left(x\left(t\right),\dot{x}\left(t\right)\right),\delta x\left(t\right)\rangle dt=0,$$

*where the curve x(t) satisfies the phenomenological constraint*

(58) $$A^{\alpha }\left(x,\dot{x}\right)\;\cdot \;\dot{x}=0,\;\;for\;\alpha =1,\ldots ,k,$$

*and for variations δx subject to the variational constraint*

(59) $$A^{\alpha }\left(x,\dot{x}\right)\;\cdot \;\delta x=0,\;\;for\;\alpha =1,\ldots ,k,$$

*with $\delta x\left(t_{1}\right)=\delta x\left(t_{2}\right)=0$.*

*This yields the system of equations*

(60) $$\left\{\begin{array}{c} \;\frac{d}{dt}\frac{\partial L}{\partial \dot{x}}-\frac{\partial L}{\partial x}-F^{\mathrm{ext}}=\lambda_{\alpha }A^{\alpha }\left(x,\dot{x}\right),\\ A^{\alpha }\left(x,\dot{x}\right)\;\cdot \;\dot{x}=0,\;\;\alpha =1,\ldots ,k. \end{array}\right.$$

*It is clear that all the variational formulations for the adiabatically closed system considered above fall into this category by appropriately choosing x, $L(x,\dot{x})$, $F^{\mathrm{ext}}\left(x,\dot{x}\right)$, and $A^{\alpha }\left(x,\dot{x}\right)$. The energy defined by $E\left(x,v\right)=\langle \frac{\partial L}{\partial v},v\rangle -L\left(x,v\right)$ satisfies $\frac{d}{dt}E=\langle F^{\mathrm{ext}},\dot{x}\rangle$.*

*The constraints involved in this variational formulation admit an intrinsic geometric description. The variational constraint (Equation (59)) defines the subset $C_{V}\subset TQ\times_{Q}TQ$ given by*

$$C_{V}=\left\{\left(x,v,\delta x\right)\in TQ\times_{Q}TQ|A^{\alpha }\left(x,v\right)\;\cdot \;\delta x=0,\;\;for\;\alpha =1,\ldots ,k\right\},$$

*so that $C_{V}\left(x,v\right):=C_{V}\cap \left(\left\{\left(x,v\right)\right\}\times T_{x}Q\right)$ is a vector subspace of $T_{x}Q$ for all (x,v)∈TQ. The phenomenological constraint (Equation (58)) defines the subset $C_{K}\subset TQ$ given by*

$$C_{K}=\left\{\left(x,v\right)\in TQ|A^{\alpha }\left(x,v\right)\;\cdot \;v=0,\;\;for\;\alpha =1,\ldots ,k\right\}.$$

*Then, one notes that the constraint $C_{K}$ can be intrinsically defined from $C_{V}$ as*

$$C_{K}=\left\{\left(x,v\right)\in TQ|\left(x,v\right)\in C_{V}\left(x,v\right)\right\}.$$

*Constraints $C_{V}$ and $C_{K}$ related in this way are called nonlinear nonholonomic constraints of thermodynamic type (see [1,64]).*

### 3.3. Open Thermodynamic Systems

The thermodynamic systems that we considered so far are restricted to the adiabatically closed cases. For such systems, interaction with the exterior is only through the exchange of mechanical work, and hence the first law for such systems reads

$$\frac{d}{dt}E=\langle F^{\mathrm{ext}},\dot{q}\rangle =P_{W}^{\mathrm{ext}}.$$

We now consider the more general case of open systems exchanging work, heat, and matter with the exterior. In this case, the first law reads

$$\frac{d}{dt}E=P_{W}^{\mathrm{ext}}+P_{H}^{\mathrm{ext}}+P_{M}^{\mathrm{ext}},$$

where $P_{H}^{\mathrm{ext}}$ is the power associated with the transfer of heat into the system and $P_{M}^{\mathrm{ext}}$ is the power associated with the transfer of matter into the system. As we recall below, the transfer of matter into or out of the system is associated with a transfer of work and heat. By convention, $P_{W}^{\mathrm{ext}}$ and $P_{H}^{\mathrm{ext}}$ denote uniquely the power associated with work and heat that is not associated with a transfer of matter. The power associated with a transfer of heat or work due to a transfer of matter is included in $P_{M}^{\mathrm{ext}}$.

In order to get a concrete expression for $P_{M}^{\mathrm{ext}}$, let us consider an open system with several ports, a=1,…,A, through which matter can flow into or out of the system. We suppose, for simplicity, that the system involves only one chemical species and denote by *N* the number of moles of this species. The mole balance equation is

$$\frac{d}{dt}N=\sum_{a=1}^{A}J^{a},$$

where $J^{a}$ is the molar flow rate *into* the system through the *a*th port so that $J^{a}>0$ indicates the flow into the system and $J^{a}<0$ indicates the flow out of the system.

As matter enters or leaves the system, it carries its internal, potential, and kinetic energy. This energy flow rate at the *a*th port is the product $E^{a}J^{a}$ of the energy per mole (or molar energy) $E^{a}$ and the molar flow rate $J^{a}$ at the *a*th port. In addition, as matter enters or leaves the system, it also exerts work on the system that is associated with pushing the species into or out of the system. The associated energy flow rate is given at the *a*-th port by $J^{a}p^{a}V^{a}$, where $p^{a}$ and $V^{a}$ are the pressure and the molar volume of the substance flowing through the *a*th port. From this, we get the expression

(61) $$P_{M}^{\mathrm{ext}}=\sum_{a=1}^{A}J^{a}\left(E^{a}+p^{a}V^{a}\right).$$

We refer, for instance, to [65,66] for the detailed explanations of the first law for open systems.

We present below an extension of the variational formulation to the case of open systems. In order to motivate the form of the constraints that we use, we first consider a particular case of simple open system, namely, the case of a system with a single chemical species *N* in a single compartment with constant volume *V* and without mechanical effects. In this particular situation, the energy of the system is given by the internal energy written as U=U(S,N), since $V=V_{0}$ is constant. The balance of moles and energy are respectively given by

$$\frac{d}{dt}N=\sum_{a=1}^{A}J^{a},\;\frac{d}{dt}U=\sum_{a=1}^{A}J^{a}\left(U^{a}+p^{a}V^{a}\right)=\sum_{a=1}^{A}J^{a}H^{a}$$

(see Equation (61)), where $H^{a}=U^{a}+p^{a}V^{a}$ is the molar enthalpy at the *a*th port and where $U^{a}$, $p^{a}$, and $V^{a}$ are, respectively, the molar internal energy, the pressure, and the molar volume at the *a*th port. From these equations and the second law, one obtains the equations for the rate of change of the entropy of the system as

(62) $$\frac{d}{dt}S=I+\sum_{a=1}^{A}J^{a}S^{a},$$

where $S^{a}$ is the molar entropy at the *a*th port and *I* is the rate of internal entropy production of the system given by

(63) $$I=\frac{1}{T}\sum_{a=1}^{A}J^{a}\left(H^{a}-TS^{a}-\mu \right),$$

with $T=\frac{\partial U}{\partial S}$ being the temperature and $\mu =\frac{\partial U}{\partial N}$ being the chemical potential. For our variational treatment, it is useful to rewrite the rate of internal entropy production as

$$I=\frac{1}{T}\sum_{a=1}^{A}\left[J_{S}^{a}\left(T^{a}-T\right)+J^{a}\left(\mu^{a}-\mu \right)\right],$$

where we define the entropy flow rate $J_{S}^{a}:=J^{a}S^{a}$ and also use the relation $H^{a}=U^{a}+p^{a}V^{a}=\mu^{a}+T^{a}S^{a}$. The thermodynamic quantities known at the *a*th port are usually the pressure $p^{a}$ and the temperature $T^{a}$, from which the other thermodynamic quantities, such as $\mu^{a}=\mu^{a}\left(p^{a},T^{a}\right)$ or $S^{a}=S^{a}\left(p^{a},T^{a}\right)$, are deduced in light of the state equations of the gas.

Here, we only show the variational formulation for a simplified case of open systems, namely, an open system with only one entropy variable and one compartment with a single species. So, the open system is a simple system. The reader is referred to [3] for the more general cases of open systems, such as the extensions of Equations (29)–(31) and (51)–(53) to open systems, as well as for the case when the mechanical energy of the species is taken into account.

The state variables needed to describe the system are (q,v,S,N)∈TQ, and the Lagrangian is a map

L:TQ×R×R→R,(q,v,S,N)↦L(q,v,S,N),

We assume that the system has *A* ports, through which species can flow out of or into the system, and *B* heat sources. As above, $\mu^{a}$ and $T^{a}$ denote the chemical potential and temperature at the *a*th port, and $T^{b}$ denotes the temperature of the *b*th heat source.

Find the curves q(t), S(t), Γ(t), Σ(t), W(t), N(t) which are critical for the *variational condition*

(64) $$\delta \int_{t_{1}}^{t_{2}}\;\left[L\left(q,\dot{q},S,N\right)+\dot{W}N+\dot{\Gamma }\left(S-\Sigma \right)\right]dt+\int_{t_{1}}^{t_{2}}\langle F^{\mathrm{ext}},\delta q\rangle dt=0,$$

subject to the *phenomenological constraint*

(65) $$\frac{\partial L}{\partial S}\dot{\Sigma }=\langle F^{\mathrm{fr}},\dot{q}\rangle +\sum_{a=1}^{A}\left[J^{a}\left(\dot{W}-\mu^{a}\right)+J_{S}^{a}\left(\dot{\Gamma }-T^{a}\right)\right]+\sum_{b=1}^{B}J_{S}^{b}\left(\dot{\Gamma }-T^{b}\right),$$

and for variations subject to the *variational constraint*

(66) $$\frac{\partial L}{\partial S}\delta \Sigma =\langle F^{\mathrm{fr}},\delta q\rangle +\sum_{a=1}^{A}\left[J^{a}\delta W+J_{S}^{a}\delta \Gamma \right]+\sum_{b=1}^{B}J_{S}^{b}\delta \Gamma ,$$

with $\delta q\left(t_{1}\right)=\delta q\left(t_{2}\right)=0$, $\delta W\left(t_{1}\right)=\delta W\left(t_{2}\right)=0$, and $\delta \Gamma \left(t_{1}\right)=\delta \Gamma \left(t_{2}\right)=0$.

We note that the variational constraint (Equation (66)) follows from the phenomenological constraint (Equation (65)) by formally replacing the time derivatives $\dot{\Sigma }$, $\dot{q}$, $\dot{W}$, $\dot{\Gamma }$ by the corresponding virtual displacements δΣ, δq, δW, δΓ and by removing all the terms that depend uniquely on the exterior, i.e., the terms $J^{a}\mu^{a}$, $J_{S}^{a}T^{a}$, and $J_{S}^{b}T^{b}$. Such a systematic correspondence between the phenomenological and variational constraints extends to open systems the correspondence for adiabatically closed systems verified in Equations (25) ⇝ (26), (30) ⇝ (31), (43) ⇝ (44), (52) ⇝ (53); see also Remarks 1 and 3. Note that the action functional in Equation (64) has the same form as that in the case of adiabatically closed systems.

Taking variations of the integral in Equation (64), integrating by parts, and using $\delta q\left(t_{1}\right)=\delta \left(t_{2}\right)=0$, $\delta W\left(t_{1}\right)=\delta W\left(t_{2}\right)=0$, and $\delta \Gamma \left(t_{1}\right)=\delta \Gamma \left(t_{2}\right)=0$ and using the variational constraint (Equation (66)), we obtain the following conditions:

(67) $$\begin{array}{cc} \delta q: & \;\frac{d}{dt}\frac{\partial L}{\partial {\dot{q}}^{i}}-\frac{\partial L}{\partial q^{i}}=F_{i}^{\mathrm{fr}}+F_{i}^{\mathrm{ext}},\;i=1,\ldots ,n,\\ \delta S: & \;\dot{\Gamma }=-\frac{\partial L}{\partial S},\\ \delta W: & \;\dot{N}=\sum_{a=1}^{A}J^{a},\\ \delta N: & \;\dot{W}=-\frac{\partial L}{\partial N},\\ \delta \Gamma : & \;\dot{S}=\dot{\Sigma }+\sum_{a=1}^{A}J_{S}^{a}+\sum_{b=1}^{B}J_{S}^{b}. \end{array}$$

By the second and fourth equations, the variables Γ and *W* are thermodynamic displacements as before. The main difference from the earlier cases is that now $\dot{S}$ and $\dot{\Sigma }$ are no longer equal. The physical interpretation of Σ is given below. From Equation (65), it follows that the system of evolution equations for the curves q(t), S(t), N(t) is defined by

(68) $$\left\{\begin{array}{c} \;\frac{d}{dt}\frac{\partial L}{\partial \dot{q}}-\frac{\partial L}{\partial q}=F^{\mathrm{fr}}+F^{\mathrm{ext}},\;\;\;\frac{d}{dt}N=\sum_{a=1}^{A}J^{a},\\ \;\frac{\partial L}{\partial S}\left(\dot{S}-\sum_{a=1}^{A}J_{S}^{a}-\sum_{b=1}^{B}J_{S}^{b}\right)\\ \;=\langle F^{\mathrm{fr}},\dot{q}\rangle -\sum_{a=1}^{A}\left[J^{a}\left(\frac{\partial L}{\partial N}+\mu^{a}\right)+J_{S}^{a}\left(\frac{\partial L}{\partial S}+T^{a}\right)\right]-\sum_{b=1}^{B}J_{S}^{b}\left(\frac{\partial L}{\partial S}+T^{b}\right). \end{array}\right.$$

The energy balance for this system is computed as

$$\frac{d}{dt}E=\underset{=P_{W}^{\mathrm{ext}}}{\underset{︸}{\langle F^{\mathrm{ext}},\dot{q}\rangle }}+\underset{=P_{H}^{\mathrm{ext}}}{\underset{︸}{\sum_{b=1}^{B}J_{S}^{b}T^{b}}}+\underset{=P_{M}^{\mathrm{ext}}}{\underset{︸}{\sum_{a=1}^{A}\left(J^{a}\mu^{a}+J_{S}^{a}T^{a}\right)}}.$$

From the last equation in Equation (68), the rate of entropy of the system is found by the equation

(69) $$\dot{S}=I+\sum_{a=1}^{A}J_{S}^{a}+\sum_{b=1}^{B}J_{S}^{b},$$

where *I* is the rate of internal entropy production given by

$$I=\underset{\mathrm{mechanical}\;\mathrm{friction}}{\underset{︸}{-\frac{1}{T}\langle F^{\mathrm{fr}},\dot{q}\rangle }}+\underset{\mathrm{mixing}\;\mathrm{of}\;\mathrm{matter}\;\mathrm{flowing}\;\mathrm{into}\;\mathrm{the}\;\mathrm{system}}{\underset{︸}{\frac{1}{T}\sum_{a=1}^{A}\left[J^{a}\left(\mu^{a}-\mu \right)+J_{S}^{a}\left(T^{a}-T\right)\right]}}+\underset{\mathrm{heating}}{\underset{︸}{\frac{1}{T}\sum_{b=1}^{B}J_{S}^{b}\left(T^{b}-T\right)}}.$$

From the last Equations (67) and (69), *we notice that $\dot{\Sigma }=I$ is the rate of internal entropy production*. The second and third terms in Equation (69) represent the entropy flow rate into the system associated with the ports and the heat sources. The second law requires I≥0, whereas the sign of the rate of entropy flow into the system is arbitrary.

**Figure 7:**
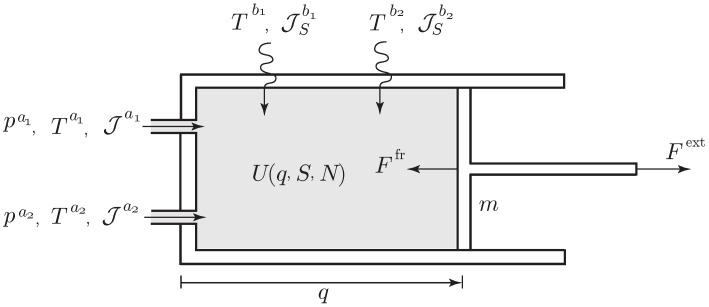
A piston device with ports and heat sources.

**Example** **5** (A piston device with ports and heat sources Figure 7)**.** *We consider a piston with mass m moving in a cylinder containing a species with internal energy U(S,V,N). We assume that the cylinder has two external heat sources with entropy flow rates $J^{b_{i}}$, i=1,2, and two ports through which the species is injected into or flows out of the cylinder with molar flow rates $J^{a_{i}}$, i=1,2. The entropy flow rates at the ports are given by $J_{S}^{a_{i}}=J^{a_{i}}S^{a_{i}}$.*

*The variable q characterizes the one-dimensional motion of the piston such that the volume occupied by the species is V=αq, with α the sectional area of the cylinder. The Lagrangian of the system is*

$$L\left(q,\dot{q},S,N\right)=\frac{1}{2}m{\dot{q}}^{2}-U\left(S,Aq,N\right).$$

*The variational formulations (Equations (64)–(66)) yield the evolution equations for q(t), S(t), N(t)*

$$m\ddot{q}=p\left(q,S,N\right)\alpha +F^{\mathrm{fr}}+F^{\mathrm{ext}},\;\dot{N}=\sum_{a=1}^{A}J^{a},\;\dot{S}=I+\sum_{i=1}^{2}J_{S}^{a_{i}}+\sum_{j=1}^{2}J_{S}^{b_{j}},$$

*where $p\left(q,S,N\right)=-\frac{\partial U}{\partial V}$ is the pressure and $I=\dot{\Sigma }$ is the internal entropy production given by*

$$I=-\frac{1}{T}F^{\mathrm{fr}}\dot{q}+\frac{1}{T}\sum_{i=1}^{2}\left[\left(\mu^{a_{i}}-\mu \right)+S^{a_{i}}\left(T^{a_{i}}-T\right)\right]J^{a_{i}}+\frac{1}{T}\sum_{j=1}^{2}J_{S}^{b_{j}}\left(T^{b_{j}}-T\right).$$

*The first term represents the entropy production associated with the friction experienced by the moving piston, the second term is the entropy production associated with the mixing of gas flowing into the cylinder at the two ports $a_{1}$, $a_{2}$, and the third term denotes the entropy production due to the external heating. The second law requires that each of these terms is positive. The energy balance holds as*

$$\frac{d}{dt}E=\underset{=P_{W}^{\mathrm{ext}}}{\underset{︸}{F^{\mathrm{ext}}\dot{q}}}+\underset{=P_{H}^{\mathrm{ext}}}{\underset{︸}{\sum_{j=1}^{2}J_{S}^{b_{j}}T^{b_{j}}}}+\underset{=P_{M}^{\mathrm{ext}}}{\underset{︸}{\sum_{i=1}^{2}\left(J^{a_{i}}\mu^{a_{i}}+J_{S}^{a_{i}}T^{a_{i}}\right)}}.$$

**Remark** **4** (Inclusion of chemical reactions)**.** *The variational formulations presented so far can be extended to include several chemical species undergoing chemical reactions. Let us denote by I=1,…,R the chemical species and by a=1,…,r the chemical reactions. Chemical reactions may be represented by*

$$\sum_{I}{\nu^{\prime }}_{I}^{a}\;I\;\overset{a_{\left(1\right)}}{\underset{a_{\left(2\right)}}{⇄}}\;\sum_{I}{\nu^{\prime \prime }}_{I}^{a}\;I,\;a=1,\ldots ,r,$$

*where $a_{\left(1\right)}$ and $a_{\left(2\right)}$ are the forward and backward reactions associated with reaction a, and ${\nu^{\prime \prime }}_{I}^{a}$, ${\nu^{\prime }}_{I}^{a}$ are the forward and backward stoichiometric coefficients for component I in reaction a. Mass conservation during each reaction is given by*

$$\sum_{I}m_{I}\nu_{I}^{a}=0\;for\;a=1,\ldots ,r\;(Lavoisier\;law),$$

*where $\nu_{I}^{a}:={\nu^{\prime \prime }}_{I}^{a}-{\nu^{\prime }}_{I}^{a}$, and $m_{I}$ is the molecular mass of species I. The affinity of reaction a is the state function defined by $A^{a}=-\sum_{I=1}^{R}\nu_{I}^{a}\mu^{I}$, a=1,…,r, where $\mu^{I}$ is the chemical potential of the chemical species I. The thermodynamic flux associated with reaction a is the rate of extent denoted $J_{a}$.*

*The thermodynamic displacements are $W^{I}$ and $\nu^{a}$ such that*

(70) $${\dot{W}}^{I}=\mu^{I},\;\;I=1,\ldots ,R\;\;\;and\;\;\;{\dot{\nu }}^{a}=-A^{a},\;\;a=1,\ldots ,r.$$

*For chemical reactions in a single compartment assumed to be adiabatically closed and without mechanical components, the variational formulation is given as follows.*

*Find the curves S(t), $N_{I}\left(t\right)$, $W^{I}\left(t\right)$, $\nu^{a}\left(t\right)$, I=1,…,R, a=1,…,r, which are critical for the variational condition*

(71) $$\delta \int_{t_{1}}^{t_{2}}\left[L\left(N_{1},\ldots ,N_{R},S\right)+{\dot{W}}^{I}N_{I}\right]dt=0,$$

*subject to the phenomenological and chemical constraints*

(72) $$\frac{\partial L}{\partial S}\dot{S}=J_{a}{\dot{\nu }}^{a}\;and\;{\dot{\nu }}^{a}=\nu_{I}^{a}{\dot{W}}^{I},\;a=1,\ldots ,r,$$

*and for variations subject to the variational constraints*

(73) $$\frac{\partial L}{\partial S}\delta S=J_{a}\delta \nu^{a}\;and\;\delta \nu^{a}=\nu_{I}^{a}\delta W^{I},\;a=1,\ldots ,r,$$

*with $\delta W^{I}\left(t_{1}\right)=\delta W^{I}\left(t_{2}\right)=0$, I=1,…,R.*

*The variational formulations (Equations (71)–(73)) yield the evolution equations for chemical reactions*

$${\dot{N}}_{I}=J_{a}\nu_{I}^{a},\;I=1,\ldots ,R\;\;\;and\;\;\;T\dot{S}=J_{a}A^{a},$$

*together with the conditions in Equation (70).*

*Chemical reactions can be included in of all the thermodynamic systems considered previously by combining the variational formulations given by Equations (71)–(73) for chemical reactions with the variational formulations given by Equations (29)–(31), (51)–(53), and (64)–(66).*

**Remark** **5** (General structure of the variational formulation for open systems)**.** *As opposed to the adiabatically closed case, the phenomenological and variational constraints depend explicitly on time t∈R for the case of open systems. In addition, the phenomenological constraint involves an affine term that depends only on the properties at the ports. From a general point of view, letting Q be the configuration manifold, these constraints are defined by the maps $A^{\alpha }:R\times TQ\to T^{*}Q$, $A\left(t,x,v\right)\in T_{x}^{*}Q$, with $A^{\alpha }\left(t,x,v\right)\in T_{x}^{*}Q$, and $B^{\alpha }:R\times TQ\to R$, α=1,…,k, where t∈R and (x,v)∈TQ.*

*Given a time-dependent Lagrangian L:R×TQ→R and an external force $F^{\mathrm{ext}}:R\times TQ\to T^{*}Q$, the variational formulations in Equations (57)–(59) are extended as follows.*

(74) $$\delta \int_{t_{1}}^{t_{2}}L\left(t,x\left(t\right),\dot{x}\left(t\right)\right)dt+\int_{t_{1}}^{t_{2}}\langle F^{\mathrm{ext}}\left(t,x\left(t\right),\dot{x}\left(t\right)\right),\delta x\left(t\right)\rangle dt=0,$$

*where the curve x(t) satisfies the phenomenological constraint*

(75) $$A^{\alpha }\left(t,x,\dot{x}\right)\;\cdot \;\dot{x}+B^{\alpha }\left(t,x,\dot{x}\right)=0,\;\;for\;\alpha =1,\ldots ,k.$$

*and for variations δx subject to the variational constraint*

(76) $$A^{\alpha }\left(t,x,\dot{x}\right)\;\cdot \;\delta x=0,\;\;for\;\alpha =1,\ldots ,k.$$

*with $\delta x\left(t_{1}\right)=\delta x\left(t_{2}\right)=0$.*

*This yields the system of equations*

(77) $$\left\{\begin{array}{c} \;\frac{d}{dt}\frac{\partial L}{\partial \dot{x}}-\frac{\partial L}{\partial x}-F^{\mathrm{ext}}=\lambda_{\alpha }A^{\alpha }\left(t,x,\dot{x}\right)\\ A^{\alpha }\left(t,x,\dot{x}\right)\;\cdot \;\dot{x}+B^{\alpha }\left(t,x,\dot{x}\right)=0,\;\;\alpha =1,\ldots ,k. \end{array}\right.$$

*The variational formulation for open systems falls into this category by appropriately choosing x and L. For instance, for Equations (64)–(66), one has x=(q,S,N,W,Γ,Σ), and L is the integrand in Equation (64). Note that in Equation (74), we chose the Lagrangian to be time-dependent for the sake of generality. In fact, all the variational formulations for thermodynamics presented above generalize easily to time-dependent Lagrangians. We refer to [3] for a full treatment.*

*The energy defined by $E\left(t,x,v\right)=\langle \frac{\partial L}{\partial v},v\rangle -L\left(t,x,v\right)$ satisfies the energy balance equation*

(78) $$\frac{d}{dt}E=\langle F^{\mathrm{ext}},\;\dot{x}\rangle -\lambda_{\alpha }B^{\alpha }-\frac{\partial L}{\partial t}.$$

*In the application to open thermodynamic systems, the first term is identified with $P_{W}^{\mathrm{ext}}$, the second term is identified with $P_{H+M}^{\mathrm{ext}}$, while the third term is due to the explicit dependence of the Lagrangian on the time.*

## 4. Variational Formulation for Continuum Thermodynamic Systems

In this section, we extend Hamilton’s principle of continuum mechanics (12) to nonequilibrium continuum thermodynamics, in the same way as Hamilton’s principle of classical mechanics (Equation (2)) was extended to the finite-dimensional case of discrete thermodynamic systems in Section 3.

We consider a multicomponent compressible fluid subject to the irreversible processes of viscosity, heat conduction, and diffusion. In presence of irreversible processes, we impose no-slip boundary conditions, hence, the configuration manifold for the fluid motion is the manifold $Q={\mathrm{Diff}}_{0}\left(D\right)$ of diffeomorphisms that keep the boundary ∂D pointwise fixed.

We assume that the fluid has *P* components with mass densities $\varrho_{A}\left(t,X\right)$, A=1,…,P in the material description, and we denote by S(t,X) the entropy density in the material description. The motion of the multicomponent fluid is given as before by a curve of diffeomorphisms $\varphi_{t}\in {\mathrm{Diff}}_{0}\left(D\right)$, but now ${\dot{\varphi }}_{t}$ is interpreted as the barycentric material velocity of the multicomponent fluid. The Lagrangian of the multicomponent fluid with irreversible processes is

$$L:T{\mathrm{Diff}}_{0}\left(D\right)\times F\left(D\right)\times F{\left(D\right)}^{P}\to R,\;\left(\varphi ,\dot{\varphi },S,\varrho_{1},\ldots ,\varrho_{P}\right)\mapsto L\left(\varphi ,\dot{\varphi },S,\varrho_{1},\ldots ,\varrho_{P}\right),$$

where F(D) denotes a space of functions on D and is given by

(79) $$\begin{array}{cc} L(\varphi ,\dot{\varphi },S,\varrho_{1},\ldots ,\varrho_{P}) & =K\left(\varphi ,\dot{\varphi },\varrho_{1},\ldots ,\varrho_{P}\right)-U\left(\varphi ,S,\varrho_{1},\ldots ,\varrho_{P}\right)\\ & =\int_{D}\left[\frac{1}{2}\varrho \left(X\right){\left|\dot{\varphi }\left(X\right)\right|}^{2}-E\left(\varrho_{1}\left(X\right),\ldots ,\varrho_{P}\left(X\right),S\left(X\right),\nabla \varphi \left(X\right)\right)\right]d^{3}X. \end{array}$$

The first term is the total kinetic energy of the fluid, where $\varrho :=\sum_{A=1}^{P}\varrho_{A}$ is the total mass density. The second term is minus the total internal energy of the fluid, where E is a general expression for the internal energy density written in terms of $\varrho_{A}\left(X\right)$, S(X), and the deformation gradient ∇φ(X). As in Equation (16), E satisfies the material covariance assumption and depends on the deformation gradient only through the Jacobian $J_{\varphi }$. As in Equation (18), there is a function ϵ, the internal energy density in the spatial representation, such that

(80) $$E\left(\varrho_{1},\ldots ,\varrho_{P},\nabla \varphi )\right)=\varphi^{*}\left[\epsilon (\rho_{1},\ldots ,\rho_{P},s)\right],\;\mathrm{for}\;\rho_{A}=\varphi_{*}\varrho_{A},\;s=\varphi_{*}S.$$

In the spatial description, the Lagrangian Equation (79) reads as

$$\ell \left(v,s,\rho_{1},\ldots ,\rho_{P}\right)=\int_{D}\left[\frac{1}{2}\rho {\left|v\right|}^{2}-\varepsilon \left(\rho_{1},\ldots ,\rho_{P},s\right)\right]d^{3}x.$$

Note that in absence of irreversible process, the Lagrangian (79) would just be defined on the tangent bundle TDiff(D) with $\varrho_{A}=\varrho_{\mathrm{ref}A}$, A=1,…,P and $S=S_{\mathrm{ref}}$ seen as fixed parameters, exactly as in Equation (15) for the single-component case.

**Remark** **6** (Material vs spatial variational principle)**.** *As we present below, the variational formulation for continuum thermodynamical systems in the material description is the natural continuum (infinite-dimensional) version of that of discrete (finite-dimensional) thermodynamical systems described in Section 3. This is analogous to the conservative reversible case recalled earlier, namely, the Hamilton principle (Equation (12)); associated with the material description of continuum systems is the natural continuum version of the classical Hamilton principle Equation (2). This is why we first consider below in Section 4.1 the variational formulation of continuum systems in the material description and deduce from it the variational formulation in the spatial description later in Section 4.2. The latter is more involved since it contains additional constraints, as we have seen in the conservative reversible case in Section 2.3.*

### 4.1. Variational Formulation in the Lagrangian Description

The variational formulation of a multicomponent fluid subject to the irreversible processes of viscosity, heat conduction, and diffusion is the continuum version of the variational formulations (Equations (51)–(53)) for finite-dimensional thermodynamic systems with friction, heat, and mass transfer. Analogous to the thermodynamic fluxes $F^{\mathrm{fr}}$, $J_{AB}$, $J^{B\to A}$ are the viscous stress, the entropy flux density, and the diffusive flux density given by $P^{\mathrm{fr}}$, $J_{S}$, $J_{A}$ in the material description. Total mass conservation imposes the condition $\sum_{A=1}^{P}J_{A}=0$.

We give below the variational formulation for a general Lagrangian with density L, i.e.,

(81) $$L\left(\varphi ,\dot{\varphi },S,\varrho_{1},\ldots ,\varrho_{P}\right)=\int_{D}L\left(\varphi ,\dot{\varphi },\nabla \varphi ,S,\varrho_{1},\ldots ,\varrho_{P}\right)d^{3}X.$$

The continuum version of the variational formulations (Equations (51)–(53)) that we propose are the following.

Find the curves φ(t), S(t), Γ(t), Σ(t), $W^{A}\left(t\right)$, $\varrho_{A}\left(t\right)$ which are critical for the *variational condition*:

(82) $$\int_{0}^{T}\;\;\int_{D}\left[L\left(\varphi ,\dot{\varphi },\nabla \varphi ,S,\varrho_{1},\ldots ,\varrho_{P}\right)+{\dot{W}}^{A}\varrho_{A}+\dot{\Gamma }\left(S-\Sigma \right)\right]d^{3}Xdt=0$$

subject to the *phenomenological constraint*

(83) $$\frac{\partial L}{\partial S}\dot{\Sigma }=-P^{\mathrm{fr}}:\nabla \dot{\varphi }+J_{S}\cdot \nabla \dot{\Gamma }+J_{A}\cdot \nabla {\dot{W}}^{A}$$

and for variations subject to the *variational constraint*

(84) $$\frac{\partial L}{\partial S}\delta \Sigma =-P^{\mathrm{fr}}:\nabla \delta \varphi +J_{S}\cdot \nabla \delta \Gamma +J_{A}\cdot \nabla \delta W^{A}$$

with $\delta \varphi \left(t_{i}\right)=\delta \Gamma \left(t_{i}\right)=\delta W^{A}\left(t_{i}\right)=0$, i=1,2, and with ${\delta \varphi |}_{\partial D}=0$.

Taking variations of the integral in Equation (82), integrating by parts, and using $\delta \varphi \left(t_{i}\right)=\delta \Gamma \left(t_{i}\right)=\delta W^{A}\left(t_{i}\right)=0$, i=1,2, and ${\delta \varphi |}_{\partial D}=0$, it follows that

∫t1t2∫D∂L∂φaδφa−∂∂t∂L∂φ˙a−∂∂A∂L∂φ,Aaδφa+∂L∂SδS+∂L∂ϱA+W˙AδϱA∂L∂φ,Aa−ϱ˙AδWA−(S˙−Σ˙)δΓ+Γ˙(δS−δΣ)d3Xdt=0.

Using the variational constraint (Equation (84)), integrating by parts, and collecting the terms proportional to δφ, δΓ, δS, $\delta W^{A}$, and $\delta \varrho_{A}$, we get

(85) $$\begin{array}{cc} \delta \varphi : & \;\frac{d}{dt}\frac{\partial L}{\partial \dot{\varphi }}+\mathrm{DIV}\left(\frac{\partial L}{\partial \nabla \varphi }+\dot{\Gamma }{\frac{\partial L}{\partial S}}^{-1}P^{\mathrm{fr}}\right)-\frac{\partial L}{\partial \varphi }=0\\ \delta \Gamma : & \;\dot{S}=\mathrm{DIV}\left(\dot{\Gamma }{\frac{\partial L}{\partial S}}^{-1}J_{S}\right)+\dot{\Sigma },\;\delta S:\;\dot{\Gamma }=-\frac{\partial L}{\partial S},\\ \delta W_{A}: & \;{\dot{\varrho }}_{A}=\mathrm{DIV}\left(\dot{\Gamma }{\frac{\partial L}{\partial S}}^{-1}J_{A}\right),\;\delta \varrho_{A}:\;{\dot{W}}^{A}=-\frac{\partial L}{\partial \varrho_{A}}, \end{array}$$

together with the boundary conditions

$$\int_{\partial D}P^{\mathrm{fr}}{}_{a}^{B}N_{B}\delta \varphi^{a}dS=0,\;\int_{\partial D}J_{S}\cdot N\delta \Gamma dS=0,\;\int_{\partial D}J_{A}\cdot N\delta W^{A}dS=0,$$

where N is the outward-pointing unit normal vector field to ∂D. The first boundary term vanishes since ${\delta \varphi |}_{\partial D}=0$ from the no-slip boundary condition. The second and third conditions give

$$J_{S}\cdot N=0\;\mathrm{and}\;J_{A}\cdot N=0,\;\;A=1,\ldots ,P,\;\mathrm{on}\;\partial D,$$

i.e., the fluid is adiabatically closed.

From the third and fifth conditions in Equation (85), we have $\dot{\Gamma }=-\frac{\partial L}{\partial S}=T$, the temperature in the material representation, and ${\dot{W}}^{A}=-\frac{\partial L}{\partial \varrho_{A}}=\Upsilon^{A}$, a generalization of the chemical potential of component *A* in the material representation. The second equation in Equation (85) thus reads as $\dot{S}+\mathrm{DIV}J_{S}=\dot{\Sigma }$ and attributes to Σ the meaning of *entropy generation rate density*. From the first and fourth equation and the constraint, we get the system

(86) $$\left\{\begin{array}{c} \;\frac{d}{dt}\frac{\partial L}{\partial \dot{\varphi }}+\mathrm{DIV}\left(\frac{\partial L}{\partial \nabla \varphi }-P^{\mathrm{fr}}\right)-\frac{\partial L}{\partial \varphi }=0\\ \;{\dot{\varrho }}_{A}+\mathrm{DIV}J_{A}=0,\;A=1,\ldots ,P\\ T\left(\dot{S}+\mathrm{DIV}J_{S}\right)=P^{\mathrm{fr}}:\nabla \dot{\varphi }-J_{S}\cdot \nabla T-J_{A}\cdot \nabla \Upsilon^{A}, \end{array}\right.$$

for the fields φ(t,X), $\varrho_{A}\left(t,X\right)$, and S(t,X). The parameterization of the thermodynamic fluxes $P^{\mathrm{fr}}$, $J_{S}$, $J_{A}$ in terms of the thermodynamic forces are discussed in the Eulerian description below.

### 4.2. Variational Formulation in the Eulerian Description

While the variational formulation is simpler in the material description, the resulting equations of motion are usually written and studied in the spatial description. It is therefore useful to have an Eulerian version of the variational formulations (Equations (82)–(84)). In order to obtain such a variational formulation, all the variables used in Equations (82)–(84) must be converted to their Eulerian analogue. We have already seen the relations $s=\varphi_{*}S$ and $\rho_{A}=\varphi_{*}\varrho_{A}$ between the Eulerian and Lagrangian mass densities and entropy densities, where the pull-back notation is defined in Equation (17). The Eulerian quantities associated with Σ, Γ, and $W^{A}$ are defined as follows

$$\sigma =\varphi_{*}\Sigma ,\;\gamma =\Gamma \circ \varphi^{-1},\;w^{A}=W^{A}\circ \varphi^{-1}.$$

The Eulerian version of the Piola–Kirchhoff viscous stress tensor $P^{\mathrm{fr}}$ is the viscous stress tensor $\sigma^{\mathrm{fr}}$ obtained via the Piola transform (see [2,48]).

From the material covariance assumption, the Lagrangian (81) can be rewritten exclusively in terms of spatial variables as

$$\ell \left(v,s,\rho_{1},\ldots ,\rho_{P}\right)=\int_{D}L\left(v,s,\rho_{1},\ldots ,\rho_{P}\right)d^{3}x,$$

where the Lagrangian density is defined by

$$L\left(v,s,\rho_{1},\ldots ,\rho_{P}\right)=\varphi_{*}\left[L(v\circ \varphi ,\varphi^{*}\rho_{1},\ldots ,\varphi^{*}\rho_{P},\varphi^{*}s)\right].$$

Using all the preceding relations between Lagrangian and Eulerian variables, we can rewrite the variational formulations Equations (82)–(84) in the following purely Eulerian form.

Find the curves v(t), s(t), γ(t), σ(t), $w^{A}\left(t\right)$, $\rho_{A}\left(t\right)$ which are critical for the *variational condition*

(87) $$\int_{0}^{T}\;\;\int_{D}\left[L\left(v,s,\rho_{1},\ldots ,\rho_{P}\right)+D_{t}w^{A}\rho_{A}+D_{t}\gamma \left(s-\sigma \right)\right]d^{3}xdt=0$$

subject to the *phenomenological constraint*

(88) $$\frac{\partial L}{\partial s}{\bar{D}}_{t}\sigma =-\sigma^{\mathrm{fr}}:\nabla v+j_{S}\cdot \nabla D_{t}\gamma +j_{A}\cdot \nabla D_{t}w^{A}$$

and for variations $\delta v=\partial_{t}\zeta +v\cdot \nabla \zeta -\zeta \cdot \nabla v$, $\delta \rho_{A}$, $\delta w^{A}$, δs, δσ, and δγ subject to the *variational constraint*

(89) $$\frac{\partial L}{\partial s}{\bar{D}}_{\delta }\sigma =-\sigma^{\mathrm{fr}}:\nabla \zeta +j_{S}\cdot \nabla D_{\delta }\gamma +j_{A}\cdot \nabla D_{\delta }w^{A}$$

with $\zeta \left(t_{i}\right)=\delta \gamma \left(t_{i}\right)=\delta w^{A}\left(t_{i}\right)=0$, i=1,2, and with ${\zeta |}_{\partial D}=0$.

In Equations (87)–(89), we use the notations $D_{t}f=\partial_{t}f+v\cdot \nabla f$, ${\bar{D}}_{t}f=\partial_{t}f+\mathrm{div}\left(fv\right)$, $D_{\delta }f=\delta f+\zeta \cdot \nabla f$, and ${\bar{D}}_{\delta }f=\delta f+\mathrm{div}\left(f\zeta \right)$ for the Lagrangian time derivatives and variations of functions and densities.

The variational formulations (Equations (87)–(89)) yield the system

(90) $$\left\{\begin{array}{c} \;\left(\partial_{t}+{£}_{v}\right)\frac{\partial L}{\partial v}=\rho_{A}\nabla \frac{\partial L}{\partial \rho_{A}}+s\nabla \frac{\partial L}{\partial s}+\mathrm{div}\sigma^{\mathrm{fr}}\\ \;{\bar{D}}_{t}\rho_{A}+\mathrm{div}j_{A}=0,\;A=1,\ldots ,P\\ \frac{\partial L}{\partial s}\left({\bar{D}}_{t}s+\mathrm{div}j_{s}\right)=-\sigma^{\mathrm{fr}}\;:\;\nabla v-j_{s}\;\cdot \;\nabla \frac{\partial L}{\partial s}-j_{A}\;\cdot \;\nabla \frac{\partial L}{\partial \rho_{A}}, \end{array}\right.$$

together with the conditions

$${\bar{D}}_{t}\sigma ={\bar{D}}_{t}s+\mathrm{div}j_{s},\;D_{t}\gamma =-\frac{\partial L}{\partial s},\;D_{t}w_{A}=-\frac{\partial L}{\partial \rho_{A}}.$$

In Equation (90), ${£}_{v}$ denotes the Lie derivative defined as ${£}_{v}m=v\cdot \nabla m+\nabla v^{T}\cdot m+m\mathrm{div}v$. We refer to [2] for a detailed derivation of these equations from the variational formulations (Equations (87)–(89)).

*The multicomponent Navier–Stokes–Fourier equations.* For the Lagrangian

$$\ell \left(v,s,\rho_{1},\ldots ,\rho_{P}\right)=\int_{D}\left[\frac{1}{2}{\rho |v|}^{2}-\epsilon (\rho_{1},\ldots ,\rho_{P},s)\right]d^{3}x$$

we get

(91) $$\left\{\begin{array}{c} \;\rho \left(\partial_{t}v+v\cdot \nabla v\right)=-\nabla p+\mathrm{div}\sigma^{\mathrm{fr}}\\ \;{\bar{D}}_{t}\rho_{A}+\mathrm{div}j_{A}=0,\;A=1,\ldots ,P\\ T\left({\bar{D}}_{t}s+\mathrm{div}j_{s}\right)=\sigma^{\mathrm{fr}}:\nabla v-j_{s}\cdot \nabla T-j_{A}\cdot \nabla \mu^{A} \end{array}\right.$$

with $\mu^{A}=\frac{\partial \epsilon }{\partial \rho_{A}}$, $T=\frac{\partial \epsilon }{\partial s}$, and $p=\mu^{A}\rho_{A}+Ts-\epsilon$.

The system of Equation (91) needs to be supplemented with phenomenological expressions for the *thermodynamic fluxes* $\sigma^{\mathrm{fr}}$, $j_{S}$, $j_{A}$ in terms of the *thermodynamic affinities* Defv, ∇T, $\nabla \mu^{A}$ compatible with the second law. It is empirically accepted that for a large class of irreversible processes and under a wide range of experimental conditions, the thermodynamic fluxes $J_{\alpha }$ are linear functions of the thermodynamic affinities $X^{\alpha }$, i.e., $J_{\alpha }=L_{\alpha \beta }X^{\beta }$, where the transport coefficients $L_{\alpha \beta }\left(\ldots \right)$ are state functions that must be determined by experiments or, if possible, by kinetic theory. Besides defining a positive quadratic form, the coefficients $L_{\alpha \beta }\left(\ldots \right)$ must also satisfy *Onsager’s reciprocal relations* [8] due to the microscopic time reversibility and the *Curie principle* associated with material invariance (see, for instance, [67,68,69,70]). In the case of the multicomponent fluid, writing the traceless part of $\sigma^{\mathrm{fr}}$ and Defv as ${\left(\sigma^{\mathrm{fr}}\right)}^{\left(0\right)}=\sigma^{\mathrm{fr}}-\frac{1}{3}\left(\mathrm{Tr}\sigma^{\mathrm{fr}}\right)\delta$ and ${\left(\mathrm{Def}v\right)}^{\left(0\right)}=\mathrm{Def}v-\frac{1}{3}\left(\mathrm{div}v\right)\delta$, we have the following phenomenological linear relations

$$-\left[\begin{array}{c} j_{S}\\ j_{A} \end{array}\right]=\left[\begin{array}{cc} L_{SS} & L_{SB}\\ L_{AS} & L_{AB} \end{array}\right]\left[\begin{array}{c} \nabla T\\ \nabla \mu^{B} \end{array}\right],\;\frac{1}{3}\mathrm{Tr}\sigma^{\mathrm{fr}}=\zeta \mathrm{div}v,\;{\left(\sigma^{\mathrm{fr}}\right)}^{\left(0\right)}=2\mu {\left(\mathrm{Def}v\right)}^{\left(0\right)}\;,$$

where all the coefficients may depend on $\left(s,\rho_{1},\ldots ,\rho_{P}\right)$. The first linear relation describes the vectorial phenomena of heat conduction (Fourier law), diffusion (Fick law), and their cross-effects (Soret and Dufour effects); the second relation describes the scalar processes of bulk viscosity with coefficient ζ≥0, and the third relation is the tensorial process of shear viscosity with coefficient μ≥0. The associated friction stress reads

$$\sigma^{\mathrm{fr}}=2\mu \mathrm{Def}v+\left(\zeta -\frac{2}{3}\mu \right)\left(\mathrm{div}v\right)\delta .$$

All these phenomenological considerations take place with the phenomenological constraint (Equation (88)) and the associated variational constraint (Equation (89)), but they are not involved in the variational condition (87). Note that our variational formulation holds independently on the linear character of the phenomenological laws.

**Remark** **7.** *For simplicity, we chose the fluid domain D as a subset of $R^{3}$ endowed with the Euclidean metric. More generally, the variational formulation can be intrinsically written on Riemannian manifolds (see [2]). Making the dependence of the Riemannian metric explicit, even if it is given by the standard Euclidean metric, is important for the study of the covariance properties [49].*

## 5. Concluding Remarks

In this paper, we survey our recent developments on the Lagrangian variational formulation for nonequilibrium thermodynamics developed in [1,2,3], which is a natural extension of Hamilton’s principle in mechanics to include irreversible processes.

Before going into details, we briefly review Hamilton’s principle as it applies to (finite-dimensional) discrete systems in classical mechanics, as well as to (infinite-dimensional) continuum systems. Then, in order to illustrate our variational formulation for nonequilibrium thermodynamics, we first start with the finite dimensional case of adiabatically closed systems together with representative examples, such as a piston containing an ideal gas, a system with a chemical species experiencing diffusion between several compartments, an adiabatic piston with two cylinders, and a system with a chemical species experiencing diffusion and heat conduction between two compartments. Then, we extend the variational formulation to open finite-dimensional systems that can exchange heat and matter with the exterior. This case is illustrated with the help of a piston device with ports and heat sources. We also demonstrate how chemical reactions can be naturally incorporated into our variational formulation.

Second, we illustrate the variational formulation with the infinite-dimensional case of continuum systems by focusing on a compressible fluid with the irreversible processes due to viscosity, heat conduction, and diffusion. The formulation is first given in the Lagrangian (or material) description because it is in this description that the variational formulation is a natural continuum extension of the one for discrete systems. The variational formulation in the Eulerian (or spatial) description is then deduced by Lagrangian reduction and yields the multicomponent Navier–Stokes–Fourier equations.

One of the key issue of our variational formulation is the introduction and the use of the concept of thermodynamic displacement, whose time derivative corresponds to the affinity of the process. Thermodynamic displacement allows for systematically developing the variational constraints associated with the nonlinear phenomenological constraints. The variational formulations presented in this paper use the entropy as an independent variable, but a variational approach based on the temperature can be also developed by considering free energy Lagrangians (see [4]).

### Further Developments

Associated with our variational formulation of nonequilibrium thermodynamics, there are the following interesting and important topics, which we have not described here due to lack of space, but they are quite relevant to the variational formulation of nonequilibrium thermodynamics, reviewed in this paper.

- *Dirac structures and Dirac systems:* It is well known that when the Lagrangian is regular, the equations of classical mechanics can be transformed into the setting of Hamiltonian systems. The underlying geometric object for this formulation is the canonical symplectic form on the phase space $T^{*}Q$ of the configuration manifold. When irreversible processes are included, this geometric formulation is lost because of the degeneracy of the Lagrangians and the presence of the nonlinear nonholonomic constraints. Hence, one may ask: what is the appropriate geometric object that generalizes the canonical symplectic form in the formulation of thermodynamics? In [64,71], it was shown that the evolution equations for both adiabatically closed and open systems can be geometrically formulated in terms of various classes of Dirac structures induced by the phenomenological constraint and from the canonical symplectic form on $T^{*}Q$ or on $T^{*}\left(Q\times R\right)$.
- *Reduction by symmetry:* When symmetries are available, reduction processes can be applied to the variational formulation of thermodynamics, thereby extending the process of Lagrangian reduction from classical mechanics to thermodynamics. This is illustrated in Section 4.2 for the Navier–Stokes–Fourier equation, but it can be carried out in general for all the variational formulations presented in this paper. For instance, we refer to [72] for the case of simple thermodynamic systems on Lie groups with symmetries.
- *Variational discretization:* Associated with the variational formulation in this paper, there exist variational integrators for the nonequilibrium thermodynamics of simple adiabatically closed systems (see [72,73]). These integrators are structure-preserving numerical schemes that are obtained by a discretization of the variational formulation. The structure-preserving property of the flow of such systems is an extension of the symplectic property of the flow of variational integrators for Lagrangian mechanics.
- *Modeling of thermodynamically consistent models:* The variational formulation for thermodynamics can be also used to derive new models, which are automatically thermodynamically consistent. We refer to [74] for an application of the variational formulation to atmospheric thermodynamics and its pseudo-incompressible approximation.
